# Anti-Inflammatory and Antiplatelet Interactions on PAF and ADP Pathways of NSAIDs, Analgesic and Antihypertensive Drugs for Cardioprotection—In Vitro Assessment in Human Platelets

**DOI:** 10.3390/medicina61081413

**Published:** 2025-08-04

**Authors:** Makrina Katsanopoulou, Zisis Zannas, Anna Ofrydopoulou, Chatzikamari Maria, Xenophon Krokidis, Dimitra A. Lambropoulou, Alexandros Tsoupras

**Affiliations:** 1 Hephaestus Laboratory, School of Chemistry, Faculty of Sciences, Democritus University of Thrace, Kavala University Campus, St. Lucas, GR-65404 Kavala, Greece; mdkatsa@chem.duth.gr (M.K.); zzannaspharmacy@gmail.com (Z.Z.); anofrid@chem.duth.gr (A.O.); 2 Hematology and Internal Medicine Departments, General Hospital of Kavala, St. Silas, GR-65500 Kavala, Greece; xatzifil@gmail.com (C.M.); kroxen1959@yahoo.gr (X.K.); 3 Laboratory of Environmental Pollution Control, Department of Chemistry, Aristotle University of Thessaloniki, GR-54124 Thessaloniki, Greece; dlambro@chem.auth.gr; 4 Center for Interdisciplinary Research and Innovation (CIRI-AUTH), Balkan Center, 10th km Thessaloniki-Thermi Rd, GR-57001 Thessaloniki, Greece

**Keywords:** cardiovascular disease, platelet aggregation, antiplatelet therapy, platelet-activating factor (PAF), adenosine diphosphate (ADP), clopidogrel, non-steroidal anti-inflammatory drugs (NSAIDs), renin–angiotensin system inhibitors (RASIs), beta-blockers, inflammation

## Abstract

Cardiovascular disease (CVD) is the leading cause of death worldwide, with pathophysiological mechanisms often involving platelet activation and chronic inflammation. While antiplatelet agents targeting adenosine diphosphate (ADP)-mediated pathways are well established in CVD management, less is known about drug interactions with the platelet-activating factor (PAF) pathway, a key mediator of inflammation. This study aimed to evaluate the effects of several commonly used cardiovascular and anti-inflammatory drug classes—including clopidogrel, non-steroidal anti-inflammatory drugs (NSAIDs), angiotensin II receptor blockers (ARBs), β-blockers, and analgesics—on platelet function via both the ADP and PAF pathways. Using human platelet-rich plasma (hPRP) from healthy donors, we assessed platelet aggregation in response to these two agonists in the absence and presence of graded concentrations of each of these drugs or of their usually prescribed combinations. The study identified differential drug effects on platelet aggregation, with some agents showing pathway-specific activity. Clopidogrel and NSAIDs demonstrated expected antiplatelet effects, while some (not all) antihypertensives exhibited additional anti-inflammatory potential. These findings highlight the relevance of evaluating pharmacological activity beyond traditional targets, particularly in relation to PAF-mediated inflammation and thrombosis. This dual-pathway analysis may contribute to a broader understanding of drug mechanisms and inform the development of more comprehensive therapeutic strategies for the prevention and treatment of cardiovascular, hypertension, and inflammation-driven diseases.

## 1. Introduction

Cardiovascular disease (CVD) remains the leading cause of mortality worldwide and is frequently associated with modifiable risk factors such as tobacco use, alcohol consumption, hypertension, hypercholesterolemia, obesity, or combinations thereof [1,2,3]. The most prevalent CVDs encountered in clinical settings include ischemic heart disease (IHD), primary and secondary cardiomyopathies, heart failure (HF), cardiac arrhythmias, and stroke. Additionally, several vascular conditions—although not primarily heart-related—are classified under CVD, including peripheral artery disease (PAD), venous thrombosis and insufficiency, aortic disease, valvular disorders, pulmonary hypertension (PH), and congenital heart diseases (CHDs). Among these, coronary heart disease (CHD)—a subset of IHD—remains the primary cause of death, followed closely by stroke. CHD most commonly presents clinically as myocardial infarction (MI) or angina pectoris (AP) [4]. While CVD is traditionally considered a disease of adulthood, its pathological origins often trace back to childhood [5], emphasizing the need for early identification and modification of risk factors—both in clinical practice and biomedical research.

Pharmacological interventions that target platelet aggregation, chronic inflammation, hypertension, and physiological stress—either individually or in combination—are essential for both the prevention and treatment of cardiovascular diseases. This study explores several drug classes with potential antiplatelet and anti-inflammatory activities: Clopidogrel, Non-Steroidal Anti-Inflammatory Drugs (NSAIDs), Antihypertensives, Beta-adrenergic blockers (β-blockers), and Analgesics. The primary aim is to evaluate their effects on platelet function, particularly their activity against both the adenosine diphosphate (ADP) and platelet-activating factor (PAF) pathways [6,7].

Platelets, or thrombocytes, are anucleate cellular fragments that play a crucial role in hemostasis—the physiological process responsible for preventing bleeding by forming stable clots at sites of vascular injury [8]. Upon such injury, platelets adhere to the exposed subendothelial matrix, become activated, and aggregate to form a platelet plug that stabilizes the damage site [9]. This aggregation is predominantly mediated by glycoprotein IIb/IIIa receptors located on the platelet surface, which facilitate cross-linking via fibrinogen binding and contribute to thrombus stabilization [10]. The mechanical and biochemical activities of glycoprotein receptors (notably GP IIb and GP IIIa) are thus critical during thrombus formation and in thrombotic disease [11]. Antiplatelet agents, therefore, are designed to inhibit platelet activation and aggregation, targeting various steps of the platelet activation cascade. Their mechanisms include inhibition of COX enzymes, antagonism of ADP and thrombin receptors, and inhibition of the GPIIb/IIIa complex [12]. Clinically, these agents are used primarily to prevent arterial thrombotic events such as myocardial infarction and ischemic stroke. Platelets contribute to the formation of hemostatic plugs at injury sites to temporarily restrict bleeding before the coagulation cascade is activated [13]. Accordingly, patients recovering from ischemic stroke or MI are routinely prescribed antiplatelet agents as part of standard secondary prevention [14,15].

Clopidogrel, a well-established ADP P2Y_12_ receptor antagonist, has demonstrated efficacy in the secondary prevention of cardiovascular events in patients with atherosclerosis [16]. Along with aspirin—a COX inhibitor—clopidogrel is one of the most widely used antiplatelet agents. While each drug is effective as monotherapy, their combination as dual antiplatelet therapy (DAPT) has shown superior efficacy in reducing the risk of thrombotic complications [17], particularly following coronary artery stent implantation. DAPT is therefore considered a safe and effective strategy in preventing acute stent thrombosis and other atherothrombotic events [18].

NSAIDs, widely used for their analgesic and anti-inflammatory properties, exert their effects primarily through inhibition of COX enzymes involved in prostaglandin synthesis [19]. They are categorized into non-selective NSAIDs, which inhibit both COX-1 and COX-2 isoforms, and selective COX-2 inhibitors (coxibs), developed to reduce gastrointestinal side effects [20]. Despite their therapeutic benefits, NSAIDs have been associated with significant cardiovascular risks. The Safety of Non-Steroidal Anti-Inflammatory Drugs (SOS) Project highlighted adverse events such as myocardial infarction, stroke, thromboembolic events, heart failure, gastrointestinal bleeding, and ulceration associated with both traditional NSAIDs and coxibs [21]. Moreover, certain NSAIDs, including ibuprofen and naproxen, demonstrate reversible antiplatelet effects that may interfere with the irreversible COX-1 inhibition conferred by aspirin. These interactions raise concerns regarding the concurrent use of NSAIDs and standard antiplatelet therapy, as they may reduce the overall efficacy of treatment and contribute to increased cardiovascular risk [22].

Antihypertensive agents, particularly angiotensin-converting enzyme inhibitors (ACEIs) and angiotensin II receptor blockers (ARBs), have also been studied for their potential antiplatelet and anti-inflammatory effects [23]. Collectively referred to as renin–angiotensin system inhibitors (RASIs), these drugs exert multiple cardioprotective effects, including enhancement of endothelial function, attenuation of vascular remodeling, and deceleration of atherosclerosis [24,25]. Clinical guidelines recommend ACEIs as first-line therapy in post-myocardial infarction care, with ARBs serving as alternatives in patients who are intolerant to ACEIs [26,27,28,29]. Although both drug classes are widely used, the comparative long-term efficacy of ACEIs versus ARBs in stable post-AMI patients remains under investigation [30,31].

Beta-blockers (BBs) are well-established in the management of ischemic heart disease and heart failure. They have consistently demonstrated mortality benefits and improved cardiovascular outcomes [32,33,34,35]. Their efficacy also extends to high-risk populations, such as patients with chronic obstructive pulmonary disease (COPD) [32,33], where their early use post-MI is associated with improved survival [36]. Beyond cardiovascular benefits, beta-blockers may also play a role in reducing all-cause mortality and hospital admissions in patients with COPD, thereby highlighting their utility in comorbid cardiopulmonary conditions [37].

Analgesics, although routinely used for pain management, can have implications for cardiovascular health. Intravenous acetaminophen has been shown to dose-dependently impair platelet function, which may raise concerns about bleeding risk, particularly in individuals with impaired hemostasis or those taking anticoagulant medications [38]. Thiocolchicoside, a semi-synthetic derivative of colchicoside with GABA-mimetic and glycinergic properties, acts as a muscle relaxant by reducing neuronal excitability at the brainstem and spinal cord levels [39,40]. While thiocolchicoside is effective in treating musculoskeletal disorders, its use in high doses—particularly in combination with sympatholytic agents like beta-blockers—may enhance autonomic effects and potentially contribute to cardiovascular disturbances, especially in patients with pre-existing cardiac conditions.

Beyond these pharmacological mechanisms, emerging evidence has identified platelet-activating factor (PAF)—chemically defined as 1-O-alkyl-2-acetyl-sn-glycero-3-phosphocholine—as a critical mediator of inflammation. PAF and its structurally related analogs are implicated in the pathogenesis of various chronic inflammatory diseases, including atherosclerosis and broader cardiovascular disorders. Furthermore, PAF-related mechanisms contribute to disease states such as renal dysfunction, cancer, malignancies, persistent infections (e.g., HIV, periodontitis, sepsis), autoimmune disorders like psoriasis, and neurodegenerative diseases [41].

In contrast, ADP serves as a central agonist in platelet activation, playing a pivotal role in thrombotic events such as myocardial infarction, stroke, and unstable angina. ADP induces platelet shape change, granule secretion, calcium mobilization, and inhibition of adenylyl cyclase, although the full identity and function of its receptor pathways remain incompletely characterized. While the majority of antiplatelet pharmacotherapy has focused on ADP-mediated pathways, there exists a substantial gap in the literature regarding drug interactions with the PAF pathway—despite its known relevance to systemic inflammation and disease progression.

To address this knowledge gap, the present study was conducted to evaluate the anti-inflammatory activity of selected pharmacological agents against the PAF-induced pathway, in addition to their antiplatelet effects against the standard platelet agonist ADP. These activities were assessed using human platelet-rich plasma (hPRP) derived from healthy blood donors [42]. Antioxidant assays were used to explore potential PAF inhibition through redox mechanisms, particularly relevant to NSAIDs and antihypertensives. Through this approach, the study aims to broaden the current understanding of how commonly used cardiovascular and anti-inflammatory drugs influence both ADP and PAF pathways and to identify new therapeutic opportunities for the prevention and management of cardiovascular and inflammation-driven diseases.

While some of the studied compounds have known effects on platelet aggregation, the novelty of this work lies in the systematic, comparative assessment of their inhibitory activity across both the ADP and PAF pathways using a unified in vitro model. The inclusion of IC_50_-based profiling and the analysis of drug combinations—especially clopidogrel-based ones—offers new insight into dual-pathway mechanisms that have not been previously explored in a consolidated manner. This dual-pathway approach highlights previously underappreciated PAF-related interactions and helps address a critical gap in the current literature, particularly in the context of cardiovascular pharmacotherapy.

## 2. Materials and Methods

### 2.1. Materials, Reagents, and Instrumentation

All pharmaceutical compounds were obtained in tablet form from licensed pharmacies and included drugs from various therapeutic classes such as analgesics (Paracetamol, Thiocolchicoside), β-blockers (Propranolol, Sotalol, Metoprolol, and Atenolol), antihypertensives (Valsartan and Candesartan), an antiplatelet agent (Clopidogrel), and NSAIDs (Ketoprofen, Naproxen, Diclofenac, Etoricoxib, Niflumic Acid, Lornoxicam, Nimesulide, and Allopurinol). The detailed physicochemical properties of the tested compounds are provided in Supplementary Table S1. All reagents and solvents used for antioxidant and platelet aggregation assays were of analytical grade and purchased from Sigma Aldrich (St. Louis, MO, USA), including 1,1-diphenyl-2-picrylhydrazyl (DPPH), 2,2′-azinobis-(3-ethylbenzothiazoline-6-sulfonic acid) (ABTS), and FRAP reagents. Ethanol and bovine serum albumin (BSA) were used for the antioxidant tests. Bovine serum albumin (BSA) was used as a physiologically relevant carrier protein to simulate plasma protein-binding conditions and to improve the solubility of lipophilic or poorly water-soluble drug compounds. Although active pharmaceutical ingredients (APIs) would offer greater consistency, the use of commercial tablet formulations reflects real-world clinical usage and availability in standard healthcare settings.

Spectrophotometric readings were conducted using an LLG-uniSPEC 2 UV-Vis spectrophotometer (LLG Labware, Meckenheim, Germany). For platelet aggregation assays, all plastic consumables and reagents were sourced from Sigma Aldrich. Blood collection tubes (evacuated S-monovettes^®^ containing sodium citrate, 20G safety needles) were obtained from Sarstedt Ltd. (Wexford, Ireland). Platelet aggregation was evaluated using a Chrono-log 490 four-channel aggregometer (Chrono-log Corp., Havertown, PA, USA), connected to AGGRO/LINK^®^8 software package. PAF, ADP, and bovine serum albumin (BSA) were also obtained from Sigma Aldrich. Centrifugation was performed with a Nahita Blue Medibas centrifuge (4000 rpm max capacity).

### 2.2. Assessment of Antioxidant Activity

The antioxidant potential of the tested pharmaceutical compounds was evaluated spectrophotometrically using three widely established assays: the 1,1-diphenyl-2-picrylhydrazyl (DPPH) radical scavenging assay, the 2,2′-azinobis-(3-ethylbenzothiazoline-6-sulfonic acid) (ABTS) assay, and the ferric reducing antioxidant power (FRAP) method, according to the protocol outlined by Papadopoulou et al. [43]. Each drug was initially pulverized manually in test tubes using a glass rod or spatula and then solubilized in 1 mL of ethanol before the addition of assay-specific reagents. To ensure the robustness of the results, all assays were also repeated using undiluted drug preparations—prepared by mixing the raw drug powder directly with ethanol in the absence of additional dilutions. The same procedure was consistently followed for all three assays (DPPH, ABTS, and FRAP) to allow direct comparison across different antioxidant mechanisms.

### 2.3. Anti-Inflammatory and Antiplatelet Activity Assessment

The anti-inflammatory activity of the drugs against the Platelet-activating factor (PAF)—pathway and the anti-platelet effect against the standard platelet agonist Adenosine 5′ Diphosphate (ADP) were evaluated in human plasma rich platelet (hPRP) from healthy blood donors, according to Tsoupras et al. [42]. Fifteen (N = 15) healthy participants were enrolled in this study based on the following inclusion criteria: absence of current medication use, confirmed healthy status, no intake of supplements or vitamin preparations in the preceding month, and comparable age range and lifestyle habits.

#### 2.3.1. Sample Preparation

Seventeen pharmaceutical drugs were individually ground into fine powders using a mortar and pestle. A single tablet from each drug was processed, and the powder was transferred into 50 mL Falcon tubes. Each tube received 3 mL of freshly prepared 2.5 mg/mL BSA solution in 0.9% NaCl. The BSA solution was prepared by dissolving 125 mg of BSA in an appropriate volume of saline to reach this concentration and mixed gently to prevent foaming. Each drug–BSA mixture was vortexed multiple times, followed by 10 min of sonication to enhance solubility. A final centrifugation at 2000 rpm for 5 min helped remove undissolved residues. The supernatants were collected and stored in aliquots at −20 °C.

#### 2.3.2. Platelet Aggregation Assays

Venous blood was collected from healthy volunteers (N = 15) in citrate-containing vacutainers at the General Hospital of Kavala, Greece. To account for biological variability, Platelet-rich plasma (PRP) from multiple donors was tested (N = at least 6 for each drug), in line with findings by Meade et al. (1985), who emphasized the significance of inter-individual differences in platelet reactivity [44]. Citrate acts by chelating ionized calcium (Ca^2+^), essential for coagulation, thus preventing clot formation upon blood sampling. It also preserves cellular integrity and stabilizes coagulation factors, making it suitable for coagulation assays such as prothrombin time (PT) and activated partial thromboplastin time (aPTT). The samples were gently handled to prevent coagulation cascade activation. Platelet-rich plasma (PRP) and platelet-poor plasma (PPP) were obtained via a two-step centrifugation protocol by the Human platelet aggregometry assay [45]:

1. **PRP Preparation**: Centrifugation at 195× *g* for 18 min at 24 °C. The PRP supernatant was aspirated using a 1000 μL automatic pipette set to 1 mL, avoiding aspiration of the underlying cellular components. Care was taken to avoid contamination with red blood cells (RBCs) and white blood cells (WBCs), which can induce hemolysis and release confounding biomolecules, compromising assay integrity.
2. **PPP Preparation**: Subsequent centrifugation of remaining PRP at 1465× *g* for 20 min at 24 °C. Only tubes with clear supernatants (no signs of hemolysis) were used for further analysis.

#### 2.3.3. Sample Loading and Instrument Setup

Clear supernatants of 250 μL Platelet-rich plasma (PRP) were used in the platelet aggregation assays. PRP samples were transferred into individual siliconized cuvettes, each containing a magnetic stir bar to ensure homogeneous mixing during the assay. The siliconized surface mimics the vascular environment, minimizing platelet activation due to surface contact. Platelet-poor plasma (PPP) was first loaded into designated cuvettes of the light transmission aggregometer to establish baseline transmission, corresponding to 0% platelet aggregation. The aggregometer’s rear slots were designated for the PPP baseline readings, while the PRP samples were loaded into the front measurement positions.

To replicate physiological conditions for IC_50_ evaluation, PRP cuvettes were pre-incubated in the rear warm slots of the instrument at 36 °C for 2–3 min. Following this, test drug solutions—thawed and equilibrated to room temperature—were added to the PRP samples. After a brief pre-incubation period (approximately 2 min), either PAF or ADP was added at defined concentrations to initiate platelet aggregation.

#### 2.3.4. Dose–Response and Inhibition Assays

For each agonist (PAF and ADP), dose–response curves were first constructed by applying increasing concentrations to PRP in the absence of inhibitors to identify the concentration required to induce approximately 50% aggregation. If no aggregation response was observed or the response plateaued, agonist concentrations were adjusted accordingly (10–50 nM of PAF and 1–10 μM ADP). Agonist solutions were vortexed thoroughly before use to ensure homogeneity.

The drug-induced inhibition assay followed a standardized procedure: test compounds were added to PRP and incubated for 2 min before re-administration of the optimal agonist dose. Platelet aggregation responses were recorded, and percent inhibition was calculated relative to the positive control (agonist alone). In cases of biphasic aggregation responses—typically observed in some donors—only the initial (primary) phase was analyzed, as the secondary wave may be confounded by platelet granule release or vesicle formation.

To ensure reproducibility, each assay was performed in triplicate, and precise pipetting techniques were employed to prevent inconsistencies. Repeated inversion mixing was used for PRP suspensions throughout, with care taken to avoid foam formation.

#### 2.3.5. Data Analysis

IC_50_ values, expressed as the concentration of drug (in µg per aggregometer cuvette) required to achieve 50% inhibition of platelet aggregation, were calculated from the inhibition curves (linear or sigmoidal fits as appropriate). A lower IC_50_ value corresponds to a higher inhibitory potency, reflecting stronger antiplatelet or anti-inflammatory activity via interference with PAF or ADP pathways. Where applicable, the “rule of three” was applied to extrapolate inhibition values from limited data points within the expected inhibition range. All data are presented as mean ± standard deviation (SD), based on a minimum of three independent experiments per drug-agonist combination (technical replicates). Biological replicates were derived from platelet-rich plasma (PRP) obtained from at least 6–10 individual donors, depending on sample availability. This design ensured both reproducibility and biological relevance.

### 2.4. Drug Combination in a 1:1 Ratio

To explore potential synergistic effects among pharmaceutical agents, a series of drug combinations were prepared in a 1:1 ratio, guided by pharmacist consultation and pharmaceutical compatibility data. Specifically, 100 μL of each drug was used per combination. The primary aim was to determine whether combining these compounds could yield enhanced biological activity, particularly by achieving lower IC_50_ values, which indicate stronger inhibitory effects on platelet aggregation, especially in the PAF (Platelet-Activating Factor) pathway.

Selection of drugs was based on three main criteria:

- Superior performance in the PAF pathway, as indicated by significantly lower IC_50_ values compared to the ADP pathway.
- Clinical relevance, with an emphasis on commonly used drugs such as Paracetamol, despite its initially high IC_50_.
- Pharmacological diversity, incorporating NSAIDs, antiplatelets, analgesics, β-blockers, and antihypertensives to assess cross-class synergism.

The tested combinations were as follows:

1. Lornoxicam + Clopidogrel (1:1)
2. Lornoxicam + Paracetamol (1:1)
3. Lornoxicam + Clopidogrel + Paracetamol (1:1:1)
4. Lornoxicam + Clopidogrel + Paracetamol + Propranolol + Candesartan (1:1:1:1:1)
5. Lornoxicam + Clopidogrel + Paracetamol + Propranolol (1:1:1:1)
6. Lornoxicam + Clopidogrel + Paracetamol + Candesartan (1:1:1:1)
7. Clopidogrel + Paracetamol (1:1)
8. Candesartan + Clopidogrel (1:1)
9. Propranolol + Clopidogrel (1:1)
10. Propranolol + Clopidogrel + Candesartan (1:1:1)
11. Propranolol + Candesartan (1:1)

These combinations were designed to assess whether co-administration enhances antiplatelet or anti-inflammatory efficacy beyond that observed in monotherapy. By comparing the IC_50_ values of these combinations to the individual drugs, we aimed to identify synergistic interactions that could potentially inform future therapeutic strategies involving multi-drug regimens.

### 2.5. Toxicological Assessment of Drug-Induced Platelet Aggregation in PRP

To investigate the potential cytotoxic or pro-aggregatory effects of various pharmaceutical compounds on human platelet function, we conducted a toxicological screening using platelet-rich plasma (PRP) isolated from healthy donors. In this in vitro assay, 100 μL of each drug solution was added directly to 250 μL PRP samples without the use of external platelet agonists, allowing for the detection of any intrinsic aggregatory activity. The mixture was incubated at 37 °C for 15 min, and aggregation responses were measured using light transmission aggregometry (LTA), a well-established technique pioneered by Born (1962), which quantifies platelet clumping through changes in optical density [46]. The experimental design followed a dose–response framework similar to that described by Thompson and Vickers (1985), enabling estimation of the half-maximal effective concentration (EC_50_) for each compound [47].

Toxicity was inferred when excessive concentrations of a drug induced platelet responses resembling pathological thrombus formation, complete platelet lysis, or aggregation via atypical pathways, indicating a disruption of normal hemostatic balance. In contrast, drugs that produced no observable change in platelet aggregation under the tested conditions were considered non-toxic to platelets in this assay.

### 2.6. Desensitization Effects and Intrinsic Platelet Activation

To assess potential desensitization effects and direct platelet activation by pharmacological agents, a secondary assay was conducted using the maximum PAF-induced aggregation response as a reference. Before the aggregation curve returned to baseline, 100 μL of either atenolol or propranolol was added to fresh platelet-rich plasma (PRP) samples obtained from separate healthy donors. Four independent experiments were performed—two with atenolol and two with propranolol. This approach allows for the evaluation of whether test compounds influence the aggregation profile independently of external agonists. Monitoring changes in the aggregation curve can help identify any intrinsic platelet-modulating effects of the drugs being tested, supporting more comprehensive pharmacological and toxicological assessments.

## 3. Results and Discussion

### 3.1. Antioxidant Activity Results and Interpretation

Although antioxidant activity was evaluated using DPPH, ABTS, and FRAP assays, the majority of tested compounds failed to demonstrate measurable or consistent activity. Most absorbance readings were aberrant or elevated, likely reflecting interference with assay chemistry rather than true antioxidant behavior. To confirm these observations, all experiments were repeated using undiluted drug suspensions. However, the results remained consistently negative, reinforcing the conclusion that the tested compounds do not possess substantial antioxidant properties.

Among the tested agents, clopidogrel presented a unique challenge: its ethanol suspension formed a turbid emulsion, leading to unstable and diffuse spectrophotometric signals that resembled particulate scattering or incomplete dissolution. Even after filtration and sonication, signal instability persisted, rendering quantitative analysis unreliable. Additionally, atenolol and several other compounds exhibited a brownish-orange hue upon ethanol solubilization, which could potentially be misconstrued as indicative of antioxidant activity. However, further spectral analysis showed no corresponding antioxidant effect, suggesting the coloration likely originated from inactive excipients or formulation components rather than the active pharmaceutical ingredients. Candesartan was the only compound that demonstrated a measurable antioxidant effect, and only in the FRAP assay—suggesting a mild reducing capacity limited to iron-based systems.

In summary, under the conditions and assay formats used in this study, the tested pharmaceutical agents generally lacked significant antioxidant activity. The findings highlight the importance of validating absorbance changes against appropriate controls to distinguish true reactivity from interference or formulation-related artifacts.

### 3.2. Anti-Inflammatory and Anti-Platelet Properties of Common Drug Classes from Data Analysis

#### 3.2.1. Data Analysis for ADP and PAF Pathway

**Figure 1:**
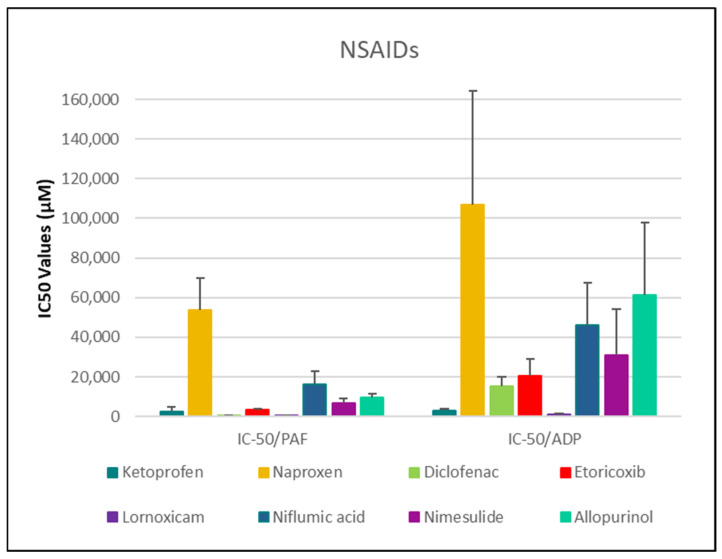
Comparative IC_50_ Values of NSAIDs Against PAF- and ADP-Induced Platelet Aggregation. This bar chart displays the mean IC_50_ values (in µM) ± standard deviation for the inhibitory effects (anti-PAF/anti-ADP activity) of various nonsteroidal anti-inflammatory drugs (NSAIDs) on platelet aggregation induced by PAF/ADP in human platelet-rich plasma (PRP). Lower IC_50_ values reflect greater anti-PAF (anti-inflammatory) or anti-ADP (antiplatelet) potency. Error bars represent standard deviation, highlighting variability across experiments. Data represent mean ± standard deviation from 6 to 10 independent experiments per drug. (The exact Mean Values and standard deviations with confidence intervals of each drug assessed against each one of the platelet agonists studied are shown in Table 1).

**Figure 2:**
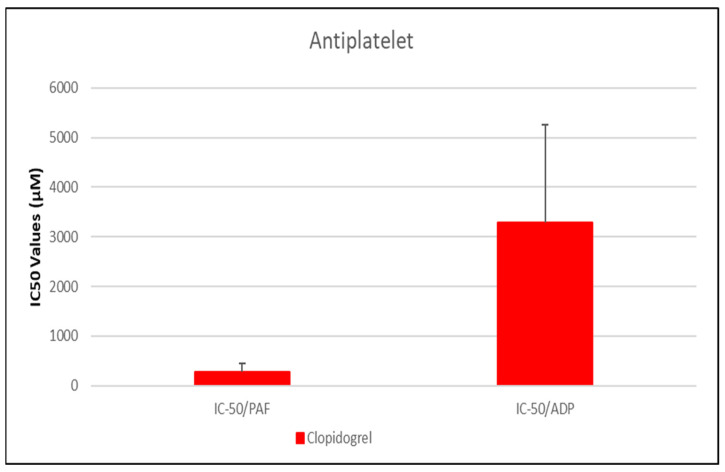
Comparative IC_50_ Values of Clopidogrel Against PAF- and ADP-Induced Platelet Aggregation. This bar graph illustrates the mean IC_50_ values (µM) ± standard deviation for clopidogrel in inhibiting platelet aggregation mediated by platelet-activating factor (PAF) and adenosine diphosphate (ADP) in human platelet-rich plasma (PRP). Clopidogrel demonstrated a markedly lower IC_50_ against PAF-induced aggregation (281.01 µM) compared to ADP-induced aggregation (3291.07 µM), indicating a stronger inhibitory effect on the PAF pathway. Error bars represent standard deviation, with greater variability observed in the ADP response. Data represent mean ± standard deviation from N = 7 independent experiments. (The exact Mean Values and standard deviations with confidence intervals of each drug assessed against each one of the platelet agonists studied are shown in Table 1).

**Figure 3:**
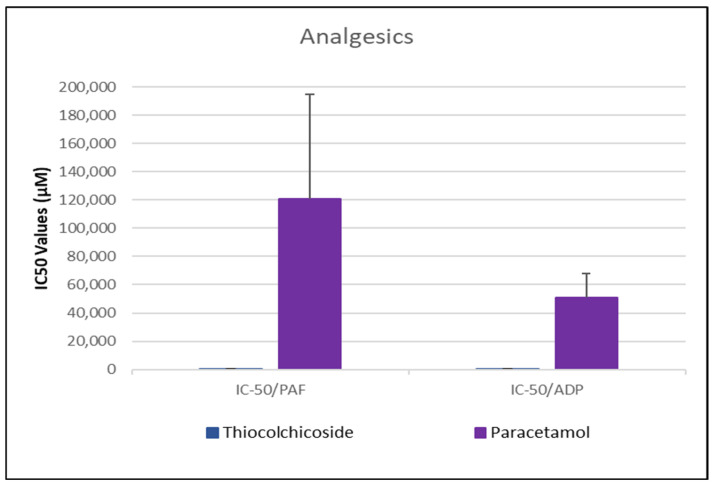
Comparative IC_50_ Values of Thiocolchicoside and Paracetamol Against PAF- and ADP-Induced Platelet Aggregation. This bar chart presents the IC_50_ values (in µM) for the inhibition of platelet aggregation by the analgesics thiocolchicoside and paracetamol in response to stimulation by platelet-activating factor (PAF) and adenosine diphosphate (ADP) in human platelet-rich plasma (PRP). Error bars represent standard deviation, reflecting variability—particularly in the response to paracetamol. Data represent mean ± standard deviation from N = 6 independent experiments per drug. (The exact Mean Values and standard deviations with confidence intervals of each drug assessed against each one of the platelet agonists studied are shown in Table 1).

**Figure 4:**
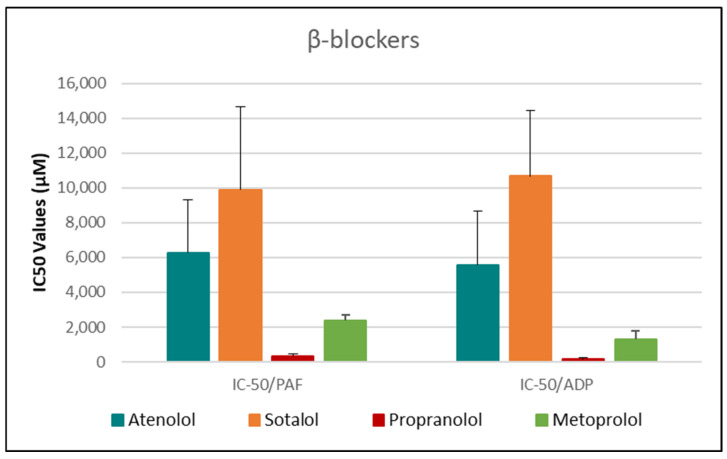
Bar chart illustrating the IC_50_ values (µM) of four β-blockers—Atenolol, Sotalol, Propranolol, and Metoprolol—on PAF- and ADP-induced platelet aggregation. This bar chart presents the mean IC_50_ values (µM) ± standard deviation for each β-blocker, reflecting their ability to inhibit platelet aggregation induced by platelet-activating factor (PAF) and adenosine diphosphate (ADP) in human platelet-rich plasma (PRP). Error bars represent standard deviations, illustrating variability in antiplatelet response across the β-blocker class. Data represent mean ± standard deviation from 6 independent experiments per drug. (The exact Mean Values and standard deviations with confidence intervals of each drug assessed against each one of the platelet agonists studied are shown in Table 1).

**Figure 5:**
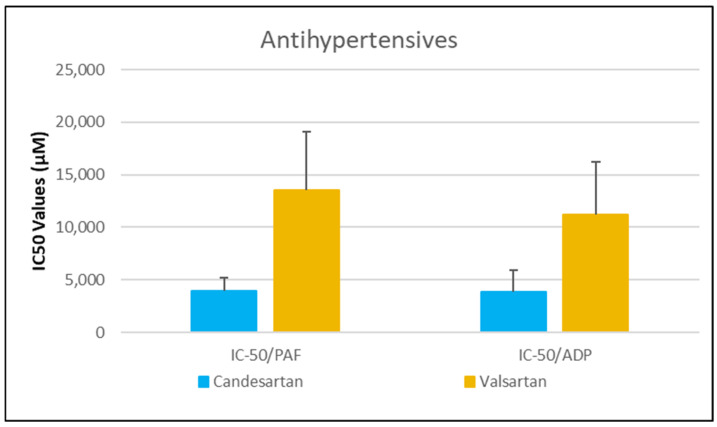
Comparative IC_50_ Values of Candesartan and Valsartan Against PAF- and ADP-Induced Platelet Aggregation. This bar chart shows the mean IC_50_ values (in µM) ± standard deviation for the inhibitory effects of two antihypertensive agents, candesartan and valsartan, on platelet aggregation induced by platelet-activating factor (PAF) and adenosine diphosphate (ADP) in human platelet-rich plasma (PRP). Error bars reflect standard deviation, highlighting variability in response between the two agents. Data represent mean ± standard deviation from 6 independent experiments per drug. (The exact Mean Values and standard deviations with confidence intervals of each drug assessed against each one of the platelet agonists studied are shown in Table 1).

As shown in Figure 1, Figure 2, Figure 3, Figure 4 and Figure 5, all tested drugs demonstrated measurable inhibitory activity against human platelet aggregation induced by platelet-activating factor (PAF), a potent inflammatory and thrombotic mediator. This inhibition was evaluated using platelet-rich plasma (PRP) from healthy donors, providing insight into each compound’s potential anti-inflammatory and anti-thrombotic effects. PAF exerts its effects via activation of the PAF receptor (PAFR), a G-protein-coupled receptor (GPCR) expressed on platelets and other immune cells, playing a central role in the pathophysiology of cardiovascular and inflammatory diseases [48]. Therefore, a drug’s ability to inhibit PAF-induced aggregation is a strong indicator of its potential to modulate thrombo-inflammatory processes. In this context, our study employed an in vitro platelet aggregation model to quantify the extent to which various commonly prescribed cardiovascular and anti-inflammatory drugs can interfere with PAF-mediated platelet activation. Notably, all drugs tested showed greater inhibitory activity against the PAF pathway than paracetamol and naproxen, with paracetamol exhibiting the weakest anti-PAF effect.

**Table 1:** Comparative IC_50_ Values (Mean ± SD), Percentage Platelet Aggregation (Mean ± SD), and 95% Confidence Intervals for Drug-Induced Inhibition of Platelet Aggregation via PAF and ADP Pathways.

Drug	IC_50_ PAF (µM)	95% CI (PAF)	% Aggr. of PAF (Mean ± SD)	IC_50_ ADP (µM)	95% CI (ADP)	% Aggr. of ADP (Mean ± SD)
Clopidogrel	281.01 ± 176.03 *	[113.6–448.4]	34.3 ± 15.5%	3291.07 ± 1961.99	[1684.4–4897.7]	44.85 ± 19.49%
Lornoxicam	313.99 ± 58.34 *	[252.8–375.2]	52.92 ± 9.04%	1276.02 ± 328.66 ^#^	[931.1–1620.9]	43.49 ± 9.61
Propranolol	346.66 ± 129.19 *	[233.1–460.2]	62.36 ± 19.45%	161.86 ± 90.22 ^#^	[85.7–238.1]	54.80 ± 25.04%
Diclofenac	531.79 ± 128.61 *	[396.8–666.8]	55.46 ± 11.02%	15,326.30 ± 4948.85	[10,132.8–20,519.8]	42.06 ± 7.80%
Thiocolchicoside	621.52 ± 201.86 *	[418.3–824.7]	52.02 ± 9.33%	405.94 ± 135.83 ^#^	[275.2–536.7]	49.10 ± 11.09%
Ketoprofen	2595.78 ± 2046.64	[1131.7–4059.9]	67.70 ± 11.13%	3080.21 ± 661.63	[2385.9–3774.5]	40.79 ± 18.69%
Metoprolol	2361.72 ± 360.88	[1995.3–2728.2]	44.63 ± 15.49%	1287.08 ± 472.37 ^#^	[906.3–1667.9]	25.47 ± 6.68%
Atenolol	6284.80 ± 3008.44	[3721.1–8848.5]	45.77 ± 19.91%	5560.11 ± 3082.81	[2938.1–8182.1]	35.33 ± 17.31%
Etoricoxib	3376.45 ± 418.77	[2937.0–3815.9]	49.89 ± 5.73%	20,625.69 ± 8629.95	[11,569.1–29,682.3]	59.87 ± 11.01%
Candesartan	3907.52 ± 1299.50	[1382.6–6432.4]	66.83 ± 9.94%	3895.13 ± 2045.13	[1963.6–5826.7]	58.43 ± 14.94%
Nimesulide	6894.14 ± 2044.11	[4962.4–8825.9]	45.50 ± 13.67%5	31,092.82 ± 22,876.90	[11,056.4–51,129.2]	56.43 ± 17.16%
Niflumic acid	16,240.62 ± 6528.41	[10,092.6–22,388.6]	58.39 ± 16.27%	45,947.12 ± 21,504.17	[23,983.2–67,911.1]	43.52 ± 16.78%
Sotalol	9901.80 ± 4750.39	[5879.6–13,923.9]	47.57 ± 20.22%	10,661.67 ± 3755.12	[7027.8–14,295.5]	24.90 ± 9.32%
Allopurinol	9634.41 ± 2110.18	[7187.1–12,081.8]	47.43 ± 9.71%	61,368.99 ± 36,384.12	[22,955.6–99,782.3]	46.06 ± 14.92%
Valsartan	13,572.57 ± 5513.50	[4550.9–22,594.2]	74.40 ± 11.54%	11,172.65 ± 5076.32	[3690.6–18,654.7]	54.16 ± 18.24%
Naproxen	53,914.04 ± 15,901.04	[39,208.0–68,620.0]	68.05 ± 6.61%	106,864.36 ± 57,249.36	[46,784.8–166,943.9]	63.18 ± 8.74%
Paracetamol	121,042.32 ± 73,625.26	[52,537.9–189,546.7]	61.51 ± 10.50%	50,720.23 ± 17,357.19	[34,694.5–66,745.9]	55.29 ± 15.53%

* denotes the compounds with lower than 1000 μM IC_50_ values against PAF and thus the most bioactive anti-PAF drugs, while ^#^ denotes the compounds with lower than 1500 μM IC_50_ values against ADP and thus the most bioactive anti-ADP drugs.

Parallel evaluation of the drugs’ anti-thrombotic potential was conducted via assessment of their inhibition of ADP-induced platelet aggregation, a classical model of platelet activation relevant to thrombotic disease. ADP acts through the P2Y_1_ and P2Y_12_ GPCRs on platelets, activating cyclooxygenase (COX) and arachidonic acid pathways, both central to thrombus formation [48]. As shown in Figure 1, Figure 2, Figure 3, Figure 4 and Figure 5, all compounds exhibited significant inhibition of ADP-induced aggregation too, although with varied potencies. Interestingly, several drugs demonstrated a significantly stronger inhibitory effect against the PAF pathway compared to the ADP pathway (*p* < 0.05), as evidenced by consistently lower IC_50_ values for PAF-induced aggregation. This selective inhibition was especially notable for naproxen, niflumic acid, nimesulide, and allopurinol, suggesting a preferential anti-inflammatory profile through PAFR interference, rather than a direct anti-platelet effect via P2Y_12_ antagonism. Conversely, paracetamol exhibited greater selectivity toward the ADP pathway, aligning more closely with a pro-thrombotic inhibitory profile.

When comparing overall efficacy, NSAIDs such as ketoprofen and lornoxicam showed the most potent anti-ADP activity, followed by diclofenac and etoricoxib, which exhibited moderate effects. Nimesulide, although more effective than naproxen, allopurinol, and paracetamol, was significantly less potent than ketoprofen and lornoxicam (*p* < 0.05), placing it in an intermediate efficacy group. Allopurinol, paracetamol, and niflumic acid consistently showed the weakest anti-ADP effects, with all three performing significantly below the majority of other drugs tested (*p* < 0.05). In the β-blocker class, atenolol, metoprolol, and propranolol displayed strong anti-ADP activity, on par with the more potent NSAIDs. Sotalol, in contrast, aligned more closely with the moderate activity group of diclofenac and etoricoxib. Among antihypertensives, candesartan matched the high efficacy of ketoprofen and lornoxicam, while valsartan grouped with the moderate inhibitors. The analgesic thiocolchicoside also demonstrated potent anti-ADP activity comparable to that of the most effective NSAIDs, unlike paracetamol, which remained among the least effective. Clopidogrel, as expected, showed one of the strongest inhibitory effects on ADP-induced aggregation, consistent with its established P2Y_12_ antagonism. Its high potency confirmed the validity of the assay and its comparative potential in evaluating unknown drugs.

Overall, these findings emphasize that:

- NSAIDs and clopidogrel generally favor the inhibition against the PAF pathway, supporting an anti-PAF specificity and thus a more profound anti-inflammatory efficacy, followed by dual anti-platelet roles.
- Analgesics and β-blockers leaned toward anti-ADP selectivity, suggesting a more antiplatelet antithrombotic—targeted effect.
- Antihypertensives exhibited balanced activity across both pathways, with slight variation depending on the specific compound.

To statistically evaluate the observed differences across drugs and between pathways (PAF vs. ADP), a one-way analysis of variance (ANOVA) was performed, followed by post hoc testing using IBM SPSS software (IBM-SPSS statistics 30 for Windows, SPSS Inc., Chicago, IL, USA). Significant differences (*p* < 0.05) confirmed distinct pathway selectivities and potencies among the drug classes. These results underscore the value of targeting multiple platelet activation pathways when assessing the anti-thrombotic and anti-inflammatory potential of pharmacological agents.

#### 3.2.2. Anti-Inflammatory and Anti-Platelet Properties of Common Drug Classes: Literature Evidence and Experimental Findings

##### NSAIDs—Diclofenac, Naproxen, Ketoprofen, Lornoxicam, Etoricoxib, Niflumic Acid, Nimesulide, and Allopurinol

With respect to NSAIDs, lornoxicam and diclofenac exhibited the strongest inhibitory effects on platelet aggregation in both pathways, while naproxen was the least effective (Figure 1). More specifically, Ketoprofen exhibited IC_50_ values of 2595.78 ± 2046.64 µM [95% CI: 1131.7–4059.9] for PAF and 3080.21 ± 661.63 µM [95% CI: 2385.9–3774.5] for ADP-induced aggregation. Naproxen showed significantly weaker activity, with IC_50_ values of 53,914.04 ± 15,901.04 µM [95% CI: 39,208.0–68,620.0] (PAF) and 106,864.36 ± 57,249.36 µM [95% CI: 46,784.8–166,943.9] (ADP). Diclofenac demonstrated strong inhibition with IC_50_ values of 531.79 ± 128.61 µM [95% CI: 396.8–666.8] (PAF) and 15,326.30 ± 4948.85 µM [95% CI: 10,132.8–20,519.8] (ADP). Etoricoxib presented IC_50_ values of 3376.45 ± 418.77 µM [95% CI: 2937.0–3815.9] (PAF) and 20,625.69 ± 8629.95 µM [95% CI: 11,569.1–29,682.3] (ADP), while lornoxicam, the most potent agent, exhibited IC_50_ values of 313.99 ± 58.34 µM [95% CI: 252.8–375.2] and 1276.02 ± 328.66 µM [95% CI: 931.1–1620.9], respectively. Niflumic acid showed moderate activity with IC_50_ values of 16,240.62 ± 6528.41 µM [95% CI: 10,092.6–22,388.6] (PAF) and 45,947.12 ± 21,504.17 µM [95% CI: 23,983.2–67,911.1] (ADP), whereas nimesulide had IC_50_ values of 6894.14 ± 2044.11 µM [95% CI: 4962.4–8825.9] and 31,092.82 ± 22,876.90 µM [95% CI: 11,056.4–51,129.2], respectively. Allopurinol displayed relatively weak inhibition with IC_50_ values of 9634.41 ± 2110.18 µM [95% CI: 7187.1–12,081.8] (PAF) and 61,368.99 ± 36,384.12 µM [95% CI: 22,955.6–99,782.3] (ADP).

I. Diclofenac

Diclofenac, a commonly used nonsteroidal anti-inflammatory drug (NSAID), exhibits both anti-inflammatory and mild antiplatelet effects primarily through cyclooxygenase (COX) inhibition. Literature consistently reports that diclofenac reduces thromboxane A_2_ (TxA_2_) synthesis by inhibiting COX enzymes, particularly COX-1, thereby impairing arachidonic acid (AA)-induced platelet aggregation [49,50].

While its effect on ADP- or collagen-induced aggregation is generally minimal in platelet-rich plasma (PRP) from NSAID users [50], some animal studies and in vitro models demonstrate broader inhibitory potential. For instance, in a rabbit model, diclofenac altered platelet-activating factor (PAF) and tumor necrosis factor (TNF) levels, implicating its role in modulating inflammatory mediators associated with platelet aggregation [51]. In Swiss albino mice, diclofenac induced hematological changes, including alterations in platelet count, which were alleviated by Olea europaea leaf extract, indicating a protective effect against diclofenac-induced hematotoxicity [52]. Additionally, Schmidt et al. showed that diclofenac significantly reduced TxA_2_ levels in human blood cell models, confirming its COX-inhibitory effects on platelet function [53]. These studies suggest a species- and model-dependent variation in diclofenac’s effects on platelet aggregation.

Diclofenac has been reported to exert variable effects on platelet function depending on context and study design. Osojnik and Kamenik (2020) found that diclofenac use in patients undergoing coronary artery bypass grafting (CABG) did not impair platelet function and was associated with lower C-reactive protein (CRP) levels, suggesting a reduced postoperative inflammatory response [54]. In a comparative study evaluating the impact of various intravenous NSAIDs, Niemi et al. observed that diclofenac exerted only a mild inhibitory effect on adrenaline-induced platelet aggregation and negligible effects on ADP pathways. These findings suggest a relatively safer platelet profile for diclofenac compared to ketoprofen and ketorolac [55]. Additionally, Shtrygol’ et al. (2023) reported that in rats exposed to cold-induced injury, diclofenac combined with etoricoxib normalized hemostatic parameters such as D-dimer, fibrinogen, and thrombin time. This suggests a stabilizing effect on systemic hemostasis under certain stress conditions [56].

Diclofenac has shown selective and potentially beneficial effects on platelet function in certain experimental contexts. Dubrall et al. [57] noted that diclofenac inhibited COX enzymes and reduced thromboxane A_2_ (TxA_2_) production, which could enhance bleeding risk, particularly in older adults, but this is more relevant to safety in high-risk populations than a direct pro-thrombotic effect. Furthermore, Dewi et al. [58] demonstrated immunomodulatory benefits of diclofenac in human PBMCs during Candida albicans infection, restoring interferon-γ (IFN-γ) levels and suppressing prostaglandin E_2_ (PGE_2_), indicating potential anti-inflammatory synergy without adverse platelet effects.

Animal studies further support antiplatelet or protective roles. Huang et al. [59] found that diclofenac reduced ADP- and PAF-induced platelet aggregation when combined with NAV2729 and EST, while Hanumegowda et al. [60] reported impaired platelet function in rats after diclofenac exposure; however, this was reversed by PEKS, highlighting a potential protective interaction. In cold-exposed rats, Shtrygol’ et al. [56] observed that diclofenac combined with etoricoxib normalized D-dimer, fibrinogen, and thrombin time, suggesting restoration of hemostatic balance under stress conditions. Finally, in KOA rat models, diclofenac was shown to reduce inflammation through platelet-related metabolic modulation, contributing to an overall anti-inflammatory state [61].

In our study, 100 mg of diclofenac dissolved in 3 mL of bovine serum albumin (BSA) inhibited PAF-induced platelet aggregation with an IC_50_ of approximately 532 µM, while ADP-induced aggregation showed a much higher IC_50_ of 15,326 µM (standard deviations: 129 µM and 4949 µM, respectively). The relatively low IC_50_ against the PAF pathway suggests significant potency, particularly when compared to the positive control, clopidogrel, a classical antiplatelet agent. This indicates a degree of pathway specificity favoring PAF inhibition.

Our findings on diclofenac’s antiplatelet effects are supported by previous literature. In particular, Andrioli et al. demonstrated that diclofenac enhanced platelet adhesion at concentrations of 100–500 μM under resting conditions but inhibited arachidonic acid–induced aggregation and increased intracellular Ca^2+^ levels via a 12-lipoxygenase–mediated mechanism—without inducing classical aggregation [62]. In line with our data, Niemi et al. reported that intravenous diclofenac (75 mg) reduced adrenaline-induced platelet aggregation to a median of 22.5% at 2 h, while showing no significant effect on ADP-induced aggregation at concentrations up to 6 μM, suggesting weak and pathway-selective inhibition [55]. Although an IC_50_ was not reported, the lack of effect on ADP responses at therapeutic concentrations implies that the IC_50_ would exceed 15,000 μM—comparable to our experimental result of 15,326 μM. Supporting this, Andrioli et al. also noted that diclofenac inhibited ADP- and thrombin-induced platelet adhesion only at concentrations above 500 μM, reinforcing a threshold-dependent, dual effect [63]. These collective findings highlight diclofenac’s moderate potency in inhibiting platelet aggregation through PAF and suggest limited efficacy in ADP-related pathways at standard dosing levels.

Overall, the stronger inhibition of the PAF pathway relative to the ADP pathway suggests potential selectivity. However, the relatively high IC_50_ for ADP indicates limited potency in that pathway. Together, these results underscore diclofenac’s multifaceted but context-dependent effects on platelet function, influenced by dosage, administration route, and the specific biological environment.

Despite these favorable findings, some studies suggest potential pro-thrombotic risks or dual effects of diclofenac. For example, Struthmann et al. (2009) demonstrated that diclofenac reduces prostacyclin (PGI_2_) without significantly altering thromboxane A_2_ (TxA_2_), leading to a pro-thrombotic imbalance that promotes platelet aggregation and accelerates thrombotic vessel occlusion [64]. Falcinelli et al. (2019) assessed ocular NSAID administration and found that, unlike indomethacin, diclofenac did not significantly affect platelet P-selectin expression or thromboxane B_2_ generation, suggesting limited COX-1 inhibition in platelets. However, this could also mean limited systemic antiplatelet efficacy when applied topically [65]. Therefore, while ocular administration of diclofenac may have limited systemic effects on platelet function, systemic administration could potentially alter the thromboxane-prostacyclin balance and platelet count, contributing to increased thrombotic risk under certain conditions.

A rare but serious adverse effect was reported by Lara et al. (2022), where diclofenac-induced thrombotic thrombocytopenic purpura (TTP) was linked to ADAMTS13 inhibition, underscoring potential hematological toxicity [66]. Another study by Andrioli et al. found that at high concentrations, diclofenac paradoxically enhanced platelet adhesion, despite inhibiting arachidonic acid-induced aggregation, illustrating a dose-dependent duality in its mechanism [62]. Thus, despite its antiplatelet properties, diclofenac may exert pro-thrombotic or adverse hematological effects in specific scenarios. Özdemir et al. [67] reported a case of diclofenac-induced Kounis Syndrome, in which platelet aggregation was enhanced via thromboxane release. Korolova et al. [68] demonstrated in an animal model that diclofenac-induced hepatitis increased platelet aggregation due to associated liver dysfunction. Similarly, Abdel-Rahman and Abdel-Baky [69] observed oxidative stress and inflammation from diclofenac exposure, which led to compensatory increases in platelet count—a potentially thrombotic state.

Moreover, Salim et al. [70] found that diclofenac led to platelet count reductions linked to liver damage and hormonal imbalance, while Basheeruddin et al. [71] identified secondary platelet dysfunction resulting from systemic toxicity, although this was mitigated by co-administration of betaine. Also, Gut et al. [72] warned that strong COX-2 inhibition by diclofenac may shift the prostacyclin-thromboxane balance toward thrombosis, raising cardiovascular risk. Additionally, in a study involving sheep, diclofenac combined with tilmicosin decreased platelet counts and elevated bleeding risk [73], further complicating its risk profile in veterinary or dual-treatment settings.

II. Naproxen

Naproxen, a widely used non-selective nonsteroidal anti-inflammatory drug (NSAID), is known for its anti-inflammatory, analgesic, and antipyretic properties. It inhibits cyclooxygenase-1 (COX-1), leading to reduced thromboxane A_2_ (TxA_2_) synthesis and impaired platelet aggregation. This mechanism increases bleeding risk, particularly in post-myocardial infarction (MI) patients receiving antithrombotic therapy [74]. Compared to selective COX-2 inhibitors, naproxen exhibits a stronger antiplatelet effect due to its broader COX inhibition.

Like ibuprofen, naproxen can interfere with the antiplatelet activity of aspirin by competing for the COX-1 binding site, potentially preventing aspirin’s irreversible acetylation of the enzyme [22]. This interaction has important clinical implications. For example, Gurbel et al. reported that while a single dose of naproxen sodium had no effect on aspirin-induced thromboxane inhibition, chronic co-administration over ten days produced a pharmacodynamic interaction that persisted for up to three days after discontinuing naproxen. These findings emphasize the importance of dosing strategy—specifically, that immediate-release aspirin should be taken at least 30 min before naproxen to mitigate this interaction [75].

In a study by Clarke et al., naproxen was evaluated in combination with GS-9876 (lanraplenib), a spleen tyrosine kinase inhibitor. Both agents independently inhibited platelet aggregation induced by convulxin and arachidonic acid (AA). However, their combination did not produce consistently additive effects across all donors. While some individuals exhibited enhanced inhibition when naproxen was combined with GS-9876 or aspirin, the response varied considerably, highlighting the inter-individual variability in drug interaction outcomes [76]. Naproxen’s antiplatelet effects are particularly evident in pathways involving AA. Several studies have shown that its inhibitory action is more pronounced in AA-induced platelet aggregation than in responses initiated by adenosine diphosphate (ADP) [77].

Nevertheless, naproxen has also demonstrated moderate inhibitory effects on ADP-induced aggregation. In a crossover study comparing naproxen with nabumetone in patients with rheumatoid arthritis, naproxen significantly reduced secondary aggregation induced by both ADP and epinephrine. This reinforces the stronger antiplatelet effect of naproxen compared to partially COX-2-selective agents like nabumetone—an important consideration in patients with elevated bleeding risk [78]. Further, studies show that naproxen inhibits the second wave of ADP-induced platelet aggregation, which reflects its effect on thromboxane A_2_-dependent signaling. This inhibition is reversible, with partial recovery observed within two days of discontinuation [79]. In vitro studies confirm that naproxen’s effect is dose-dependent and comparable to aspirin in potency, likely mediated through inhibition of prostaglandin and thromboxane synthesis [80].

In experimental conditions, naproxen at 500 mg in 3 mL BSA exhibited moderate inhibition of platelet aggregation induced by both PAF and ADP. Reported IC_50_ values were approximately 53,914 μM for PAF and 106,864 μM for ADP, suggesting slightly greater sensitivity in the PAF pathway. However, these values are much higher than typical IC_50_ values for COX-1 inhibition, indicating that naproxen’s potency in this system may be limited, possibly due to assay conditions or protein binding variability (SD_PAF_ ≈ 15,901 μM; SD_ADP_ ≈ 57,249 μM).

Overall, naproxen’s effects on platelet function are complex and influenced by multiple factors, including dosage, duration of use, timing relative to other medications (especially aspirin), and individual biological variability. These findings underscore the need for careful therapeutic planning when naproxen is used in patients at risk for cardiovascular events or requiring antiplatelet therapy.

III. Ketoprofen

Ketoprofen, a widely used nonsteroidal anti-inflammatory drug (NSAID), exhibits significant antiplatelet activity through non-selective inhibition of cyclooxygenase (COX), particularly COX-1. This inhibition reduces thromboxane A_2_ (TxA_2_) synthesis, thereby impairing platelet aggregation. It is commonly prescribed for musculoskeletal pain and arthritis due to its affordability and over-the-counter availability [81].

In human studies, extended-release ketoprofen (200 mg daily) has been shown to inhibit platelet aggregation by over 50%, with reductions in TxB_2_ production of up to 85%. Notably, this effect persisted even with concurrent aspirin use, suggesting substantial independent antiplatelet activity [82]. Another study demonstrated that ketoprofen dose-dependently inhibited ADP-induced platelet aggregation by up to 85% and suppressed TxB_2_ formation by as much as 97%, highlighting its potent COX-1 inhibition [83]. In addition, although human data are limited, ketoprofen has been shown to inhibit PAF-induced platelet aggregation in bovine models, suggesting possible activity on PAF-mediated pathways [84].

In contrast, other studies report a more selective profile. For example, while ketoprofen significantly prolonged bleeding time, it did not notably inhibit ADP-induced platelet aggregation in healthy volunteers, indicating a more limited effect on ADP pathways [85]. Further evidence from high-dose ketoprofen administration (600 mg initially, followed by 200 mg every 8 h for seven days) confirmed a marked suppression of ADP-induced platelet aggregation, with recovery within 36 h post-treatment. Bleeding time increased modestly, but prothrombin activity and partial thromboplastin time remained stable. These findings reinforce the drug’s potent but reversible effect on platelet function [86].

In veterinary studies, ketoprofen has demonstrated similar effects. In canine models, it reduced ADP- and epinephrine-induced aggregation in platelet-rich plasma without affecting shape change, although PFA-100 closure times increased, indicating impaired function [87,88]. However, responses varied by context. For instance, in dogs treated for osteoarthritis, platelet aggregation time was unaffected [89], whereas in surgical models, collagen-induced aggregation was reduced without affecting mucosal bleeding time [90]. In cats, both IV and oral ketoprofen administration decreased serum TxB_2_ levels, consistent with COX-1 inhibition [91].

In humans, ketoprofen and related NSAIDs reduce TxB_2_ production in both platelets and mononuclear cells, reflecting systemic COX inhibition [92]. Despite these benefits, ketoprofen has been linked to thrombocytopenia. Razi et al. hypothesized that this may stem from LDH inhibition in platelets, impairing energy metabolism and activation, and recommended platelet monitoring in at-risk patients [93]. More recently, novel NSAID–carbonic anhydrase hybrids based on ketoprofen showed anti-inflammatory activity without affecting platelet aggregation or TxB_2_ production, suggesting potential for safer NSAID design [94].

Our experimental findings reinforce and expand upon existing literature regarding the antiplatelet effects of ketoprofen, while highlighting the critical influence of assay conditions. In BSA-based in vitro assays, ketoprofen (25 mg in 3 mL BSA) exhibited moderate inhibition of platelet aggregation, with IC_50_ values of approximately 2596 μM for PAF-induced aggregation and 3080 μM for ADP-induced aggregation (standard deviations: 2047 μM and 662 μM, respectively). Although overall inhibitory activity was modest, the slightly lower IC_50_ against PAF suggests a minor but discernible pathway preference for PAF-mediated activation.

In contrast, a clinical crossover study reported that intravenous ketoprofen (1.4 mg/kg) significantly reduced ADP-induced platelet aggregation at 6 μM ADP, with median maximal aggregation falling to 18.3% at 2 h post-infusion and an estimated IC_50_ of approximately 5–10 μM [55]. The discrepancy between the clinical and experimental IC_50_ values can be attributed to several factors: the route of administration (intravenous vs. in vitro), the presence of BSA in our assays—which binds strongly to ketoprofen and reduces the free drug concentration—and the higher overall drug levels used in vitro.

Taken together, ketoprofen demonstrates intermediate antiplatelet potency, with a modest selectivity toward PAF-induced aggregation, and its effectiveness is heavily influenced by pharmacokinetic context, protein binding, and experimental design.

IV. Lornoxicam

Lornoxicam, another NSAID, has demonstrated a notable capacity to inhibit platelet aggregation. Specifically, Blaicher et al. (2004) demonstrated that lornoxicam, like aspirin and diclofenac, significantly inhibited CD62P (P-selectin) expression on platelets stimulated with arachidonic acid and collagen, indicating suppression of platelet activation [95]. Furthermore, the same group showed that lornoxicam significantly prolonged ADP-induced PFA-100 closure times at 3 and 12 h, reinforcing its inhibitory effect on ADP-mediated aggregation [96]. Although Tsakiridis et al. discussed lornoxicam in the context of cardiopulmonary bypass and inflammation, they did not provide direct data on PAF-induced platelet aggregation [97]. Likewise, while Berg et al. confirmed that lornoxicam inhibits IL-6, nitric oxide, and COX enzymes in vitro, they did not evaluate its effect on ADP or PAF pathways directly [98].

In the present study, using 8 mg of lornoxicam dissolved in 3 mL of bovine serum albumin (BSA), we observed a potent inhibitory effect on platelet aggregation. The IC_50_ for PAF-induced aggregation was approximately 314 μM (SD ≈ 58.34), while the IC_50_ for ADP-induced aggregation was substantially higher at 1276 μM (SD ≈ 328.66). These findings indicate that lornoxicam exhibits a marked preference for the PAF pathway, making it the most effective NSAID tested in our study via this mechanism. The PAF-pathway IC_50_ was notably close to that of clopidogrel—used as a positive control—suggesting comparable efficacy. As few studies have examined NSAIDs’ direct effects on PAF-induced platelet aggregation, our data provide novel insight into lornoxicam’s mechanistic specificity.

Supporting this, clinical data confirm lornoxicam’s antiplatelet properties through other pathways. In one study, lornoxicam administration resulted in an 85% reduction in ADP-induced aggregation within 15 min, an effect that lasted at least 8 h [99]. Another investigation in pediatric patients receiving 8 mg intravenously demonstrated significant reductions in platelet aggregation induced by ADP (–45.37%), collagen (–37.04%), and arachidonic acid (–36.31%) at 2 h post-dose, with function returning to baseline by 24 h. Importantly, these effects occurred without bleeding complications, indicating a reversible and clinically safe antiplatelet profile [100]. Taken together, these findings highlight lornoxicam’s dual inhibitory capacity, with our study uniquely positioning it as a potent PAF-pathway inhibitor in vitro, warranting further investigation in thromboinflammatory settings.

V. Etoricoxib

Etoricoxib is a highly selective COX-2 inhibitor that, unlike non-selective NSAIDs, does not significantly impact platelet function. Multiple studies have shown that etoricoxib does not interfere with the antiplatelet effects of low-dose aspirin, as evidenced by stable serum thromboxane B_2_ levels and unaltered platelet aggregation responses [101]. Although etoricoxib can reduce platelet COX-1 activity at high concentrations (IC_50_ ≈ 162 µM), this does not translate into meaningful inhibition of platelet aggregation in clinical settings [102]. Preclinical studies, including rat models with bone trauma, further support this observation, revealing no significant alterations in platelet aggregation following etoricoxib administration [103]. With a COX-1/COX-2 IC_50_ ratio of approximately 344, etoricoxib strongly favors COX-2 inhibition while preserving COX-1-mediated thromboxane A_2_ production, thereby maintaining normal platelet aggregation and minimizing bleeding risk compared to non-selective NSAIDs. While rare cases of severe thrombocytopenia and thrombotic thrombocytopenic purpura have been reported [104], these appear to be idiosyncratic and not related to routine platelet inhibition.

In the present study, the effects of 90 mg etoricoxib in 3 mL BSA on platelet-rich plasma (PRP) were evaluated, yielding an IC_50_ of 3376.45 µg/mL for PAF-induced aggregation and 20,625.69 µg/mL for ADP-induced aggregation, with standard deviations of 418.77 µg/mL and 8629.95 µg/mL, respectively. These results indicate a moderate inhibitory effect on PAF-mediated platelet aggregation and a markedly weaker effect on ADP-mediated pathways, consistent with the view that etoricoxib does not significantly impair platelet function. However, given the limited number of direct experimental studies assessing etoricoxib’s effects on platelet activity—particularly in PRP-based models—further research is warranted to fully characterize its antiplatelet profile under various physiological and pathological conditions, especially in relation to the PAF pathway, where etoricoxib demonstrated notable specificity.

VI. Niflumic Acid

Niflumic acid, a nonsteroidal anti-inflammatory drug (NSAID), is known to inhibit both phospholipase A_2_ and COX-2, thereby contributing to its anti-inflammatory and analgesic properties [105]. Although direct studies on its antiplatelet effects are limited, several mechanisms suggest a possible inhibitory influence on platelet function. Notably, niflumic acid has been shown to block calcium-activated chloride channels, leading to reduced intracellular calcium influx, a critical step in platelet activation and aggregation [106]. Additionally, a patent source indicates that niflumic acid inhibits prostaglandin synthesis, further contributing to suppressed platelet aggregation [107]. While not directly studied in platelets, research published in The Journal of Physiology demonstrated that niflumic acid alters calcium signaling in vascular smooth muscle by releasing calcium from intracellular stores—highlighting its broader impact on calcium-dependent cellular processes, which are essential for platelet activation [108].

In our current study, we evaluated the effects of 250 mg niflumic acid in 3 mL BSA on platelet-rich plasma (PRP). The results revealed an IC_50_ of 16,240.62 μM for PAF-induced aggregation and 45,947.12 μM for ADP-induced aggregation, with standard deviations of 6528.41 μM and 21,504.17 μM, respectively. These extremely high IC_50_ values indicate very weak inhibitory activity, suggesting that while niflumic acid may modulate ion channels involved in platelet activation, it is not an effective direct antiplatelet agent at physiologically relevant concentrations. Taken together, the literature and our findings suggest that niflumic acid’s influence on platelets is mechanistically plausible but pharmacologically limited in potency under the tested conditions.

VII. Nimesulide

Nimesulide exhibits dual, concentration-dependent effects on platelet function. In a study using human platelet-rich plasma (PRP) from healthy donors, it inhibited adrenaline-induced platelet aggregation and thromboxane A_2_ (TxA_2_) formation at higher concentrations (1–100 μM), with an IC_50_ for TxA_2_ inhibition of approximately 1 μM. Conversely, at lower concentrations (0.01–0.1 μM), nimesulide enhanced platelet aggregation in response to subthreshold doses of adrenaline, highlighting a biphasic impact on platelet reactivity [109]. The study by Uemura et al. (2006) investigated the effects of YM-254890, a selective Gαq/11 inhibitor, on platelet functions using washed platelets from cynomolgus monkeys. YM-254890 significantly inhibited ADP-induced responses, including intracellular Ca^2+^ elevation (IC_50_ = 0.92 ± 0.28 μM) and P-selectin expression (IC_50_ = 0.51 ± 0.02 μM). ADP-induced platelet aggregation was also attenuated with an IC_50_ of <1 μM, although the study did not assess PAF-induced platelet activation [110]. Pharmacological inhibition of PLC and MAPK pathways further reduced aggregation, highlighting their role in the synergistic response [111].

In this study, 100 mg of nimesulide in 3 mL BSA exhibited greater inhibitory potency against PAF-induced platelet aggregation (IC_50_ ≈ 6894 μM ± 2044 SD) compared to ADP-induced aggregation (IC_50_ ≈ 31,093 μM ± 22,877 SD). These results suggest a preferential effect of nimesulide on the PAF pathway, consistent with prior studies describing its role in inhibiting PAF synthesis in immune cells. Specifically, nimesulide was shown to suppress PAF production in serum-treated zymosan (STZ)-activated human neutrophils, with IC_50_ values ranging from 10 to 20 μM, an effect attributed to elevated intracellular cAMP and inhibition of phospholipase A_2_ activity [112,113].

Furthermore, the synergistic effect of PAF and adrenaline on platelet aggregation has been well documented. In a human PRP model, subthreshold concentrations of PAF (5–8 nM) and adrenaline (0.5–2 μM) triggered synergistic platelet aggregation, which was inhibited in a dose-dependent manner by both COX-1 inhibitors—indomethacin (IC_50_ = 0.25 μM) and flurbiprofen (IC_50_ = 0.7 μM)—and the COX-2 inhibitor nimesulide (IC_50_ ≈ 26 μM). These findings reinforce the idea that nimesulide modulates PAF-mediated pathways through both PAF synthesis inhibition and downstream platelet signaling interference.

However, the higher IC_50_ values observed in our PRP-based BSA system—particularly compared to literature values in the low micromolar range—likely reflect experimental limitations, including high protein binding, drug sequestration in BSA, and reduced bioavailability in vitro. Despite these factors, the preferential inhibition of PAF over ADP aggregation supports the pathway-selective antiplatelet potential of nimesulide.

VIII. Allopurinol

Allopurinol, primarily used for managing gout, has been associated with cardiovascular benefits, likely due to its antioxidant and endothelial-protective effects rather than direct antiplatelet action [114]. Although some reports suggest an interaction with anticoagulants like warfarin, indicating a potential influence on hemostasis, the available evidence does not support a significant role for allopurinol in modulating platelet function. For example, studies on hyperuricemic individuals and gout patients found no meaningful changes in platelet aggregation in response to ADP, adrenaline, or collagen, even after serum uric acid levels were reduced with allopurinol treatment [115,116]. Furthermore, while animal models have demonstrated that allopurinol can mitigate PAF-induced intestinal injury by inhibiting xanthine oxidase and neutrophil infiltration, these effects appear to act on inflammatory pathways rather than platelet-mediated mechanisms [117].

In our experimental results using 100 mg allopurinol in 3 mL BSA, we observed moderate inhibitory effects on platelet aggregation, with IC_50_ values of approximately 9634 μM (±2110 SD) for PAF-induced and 61,369 μM (±36,384 SD) for ADP-induced aggregation. These values are significantly higher than those typically reported for known antiplatelet agents, supporting the literature’s consensus that allopurinol does not exert a strong antiplatelet effect. The greater sensitivity to PAF versus ADP in our results may reflect allopurinol’s known modulation of oxidative and inflammatory pathways rather than a direct platelet inhibitory mechanism.

Among the NSAIDs tested, lornoxicam and diclofenac exhibited the strongest inhibitory effects on both PAF- and ADP-induced platelet aggregation, with IC_50_ values in the low micromolar range, suggesting potent dual anti-inflammatory and antiplatelet activity. Ketoprofen showed intermediate potency, while etoricoxib demonstrated moderate inhibition, especially against PAF, consistent with its COX-2 selectivity. Nimesulide and niflumic acid displayed preferential inhibition of the PAF pathway but required higher concentrations, indicating lower overall potency. Allopurinol and naproxen showed the weakest antiplatelet activity, particularly against ADP-induced aggregation. These results support a mechanistic trend in which NSAIDs exhibit greater efficacy against PAF-mediated aggregation, reflecting their anti-inflammatory action, with considerable variability in their antithrombotic potential.

IX. Antiplatelet Agent—Clopidogrel as a Positive Control

Clopidogrel has been extensively studied across multiple cardiovascular disease (CVD) contexts. It is a first-line antiplatelet therapy for secondary prevention in ischemic stroke, reducing recurrence events to 4.3% with a favorable safety profile marked by a 5.5% bleeding incidence, making it suitable for long-term use in patients at higher bleeding risk or intolerant to stronger agents [118]. In patients undergoing intravascular ultrasound (IVUS)-guided percutaneous coronary intervention (PCI), clopidogrel demonstrated reduced bleeding risk compared to other antiplatelets, supporting its use in low-ischemic-risk and acute coronary syndrome (ACS) patients [119]. When used in dual antiplatelet therapy (DAPT) with low-dose aspirin, clopidogrel effectively reduced stent thrombosis and improved long-term cardiovascular outcomes [120]. Its antiplatelet efficacy was further validated in 60 high-risk CVD patients through P2Y12 receptor inhibition [121]. In the OATS study, clopidogrel monotherapy reduced von Willebrand factor (VWF) levels and increased the VWFpp/VWF ratio, enhancing platelet function metrics, particularly in combination with aspirin [122]. In peripheral artery disease (PAD) patients, clopidogrel lowered thrombus formation and myocardial infarction (MI) risk by reducing platelet aggregation [123]. However, variability in response was observed post-lower extremity revascularization, with some patients exhibiting high platelet reactivity, suggesting a need for personalized therapy adjustments [124].

In chronic heart disease (CHD) patients with renal impairment or improper dosing, DAPT with aspirin and clopidogrel increased bleeding risk, emphasizing individualized therapy strategies. Franczyk-Skóra et al. highlighted that DAPT reduces thrombus formation by inhibiting both the COX and ADP pathways [125]. In coronary artery disease (CAD) patients, especially those undergoing PCI and with ACS, clopidogrel reduced thrombotic risks, as shown in Liu et al.’s 2013 observational study examining mortality and MACCE outcomes [126]. Additionally, clopidogrel inhibited platelet activation via the SR-PSOX/CXCL16–CXCR6 axis, particularly in patients with elevated CXCL16 levels. Cebo et al. demonstrated that targeting the CXCR7 pathway reduced platelet activation and proinflammatory cytokines while increasing intraplatelet 12-HETrE, enhancing clopidogrel’s effects [127]. Clopidogrel’s antiplatelet effect was also observed in vitro through P2Y12 receptor blockade, especially when combined with aspirin [128]. In patients with narrowed coronary arteries, its use alongside acetylsalicylic acid reduced thrombotic events, although platelet-activating factor (PAF)-linked depressive symptoms persisted [129]. Genetic variability also affects clopidogrel efficacy; CYP2C19 loss-of-function alleles significantly reduce its effectiveness, as emphasized by Lim et al. [130].

In atrial fibrillation (AF) patients undergoing PCI, clopidogrel, particularly when combined with dabigatran in dual antithrombotic therapy (DAT), reduced ischemic risks like MI and stent thrombosis, with lower bleeding risk than triple therapy, per the RE-DUAL PCI trial [131]. Continued clopidogrel use in PCI patients significantly reduced thrombotic events, with predictive models showing early discontinuation doubles the risk of stent thrombosis [132]. A case study of a 75-year-old male post-PCI confirmed clopidogrel’s efficacy in preventing in-stent thrombosis [133]. During the COVID-19 pandemic, its use in PCI and ACS patients also reduced thrombotic events, including MI and stent thrombosis, complementing LMWH in DAPT regimens [134].

Animal studies also demonstrate clopidogrel’s efficacy. In cats with thromboembolic disease, clopidogrel reduced arterial thromboembolism recurrence to 16.7% and achieved 100% inhibition of ADP-mediated aggregation in healthy cats [135]. In rabbits, bleeding risk, including intracerebral hemorrhage, was evaluated through tongue bleeding time tests to balance efficacy and safety [136].

A reverse translational study by Jiang et al. found that clopidogrel’s inhibition of ADP-induced platelet aggregation and integrin αIIbβ3 activation was significantly reduced in mice fed a high-fat diet (HFD) compared to those on a normal diet (ND), indicating decreased pharmacodynamic response and potentially increased IC_50_ in obese models. This mirrors clinical observations of reduced clopidogrel responsiveness in overweight patients [137].

While clopidogrel is well-established for ADP-P2Y_12_ inhibition, its limitations—both in terms of unexplored mechanisms and variable clinical efficacy—are increasingly recognized in the literature. Although clopidogrel effectively inhibits the ADP-P2Y12 pathway, its influence on alternative activation pathways like PAF remains underexplored. PAF is a potent activator involved in thrombosis and inflammation [138,139], but no studies report clopidogrel’s IC_50_ against PAF-induced aggregation. This is notable given our findings that clopidogrel exhibited a lower IC_50_ for PAF-induced aggregation compared to ADP, suggesting a potentially stronger effect via this pathway. Furthermore, clopidogrel’s interaction with chemokine signaling (e.g., CXCL16, CXCR7) lacks comprehensive research. Genetic factors, particularly CYP2C19 loss-of-function alleles, also limit clopidogrel’s efficacy. For example, Black patients with IM/PM CYP2C19 genotypes post-PCI showed reduced response, confirming the need for genotype-guided therapy [140,141]. High platelet reactivity (HPR) has also been observed in PCI patients on DAPT with clopidogrel and aspirin, supporting personalized approaches [142]. Prasugrel and ticagrelor may offer more consistent inhibition across genetic profiles, as shown in comparative studies including Lim et al. [130].

In our study, we assessed the inhibitory potency of clopidogrel on platelet aggregation in human platelet-rich plasma (PRP) using a 75 mg dose and 3 mL of BSA buffer. Clopidogrel was selected as a positive control due to its well-established role as a classic antiplatelet drug, allowing us to benchmark the relative efficacy of various pharmacological classes under investigation. The IC_50_ for platelet-activating factor (PAF)-induced aggregation was 281.01 ± 176.03 μM [95% CI: 113.6–448.4], while for ADP-induced aggregation it was 3291.07 ± 1961.99 μM [95% CI: 1684.4–4897.7] (Figure 2), indicating significantly stronger inhibitory potency against the PAF pathway under our experimental conditions.

These IC_50_ values are notably higher than those reported in previous studies that used either the active metabolite of clopidogrel or experimental models incorporating in vivo metabolic activation. For example, Savi et al. (2000) reported potent inhibition of ADP-induced aggregation in washed human platelets, with IC_50_ values ranging from 0.8 to 1 μM when applying the active thiol metabolite directly [143]. Similarly, Gurbel et al. (2003) observed an IC_50_ of approximately 1–2 μM in patients receiving a 300 mg clopidogrel loading dose prior to percutaneous coronary intervention (PCI), where hepatic metabolism produces the active metabolite in vivo [144]. Weber et al. (1999) further confirmed this requirement, showing clopidogrel inhibited ADP-induced aggregation in washed human platelets with an IC_50_ of 1.9 ± 0.3 μM but had no significant effect in PRP unless metabolically activated [145].

The substantially higher IC_50_ values observed in our study likely stem from several experimental differences: the lower clopidogrel dose used, the lack of metabolic conversion in vitro to its active thiol metabolite, and the use of PRP, which contains plasma proteins that may sequester the drug or interfere with receptor binding. Unlike washed platelet preparations or in vivo models, our system does not replicate the hepatic bioactivation that is essential for clopidogrel’s efficacy. These factors collectively reduce the apparent pharmacodynamic effect of clopidogrel in our assay, explaining the elevated IC_50_ values compared to studies employing direct application of the active form or higher-dose in vivo protocols.

Despite the relatively robust inhibition we observed against the PAF pathway, it is noteworthy that no published studies to date have reported the IC_50_ of clopidogrel for PAF-induced aggregation. This represents a meaningful gap in the literature and underscores the potential value of our findings in characterizing clopidogrel’s broader antiplatelet effects across multiple signaling pathways.

Clopidogrel demonstrated a notably stronger inhibitory effect against PAF-induced platelet aggregation compared to ADP-induced responses in our in vitro PRP model, with IC_50_ values of 281.01 μM vs. 3291.07 μM, respectively. This contrasts with the classical understanding of clopidogrel as a P2Y_12_ antagonist primarily targeting the ADP pathway. While its elevated IC_50_ values in our study are likely due to the lack of hepatic bioactivation and protein binding in PRP, the unexpected potency against PAF suggests a potential underrecognized mechanism of action. These findings highlight the importance of pathway-specific analysis and underscore the utility of clopidogrel as a pharmacological benchmark, especially when comparing novel or repurposed agents targeting non-ADP pathways. The data also emphasize the need for further investigation into clopidogrel’s potential off-target or PAF-related effects, particularly given the absence of prior IC_50_ data for this pathway in the literature.

##### Analgesics—Thiocolchicoside and Paracetamol

With respect to analgesic drugs, Thiocolchicoside demonstrated strong inhibitory activity, with IC_50_ values of 621.52 ± 201.86 µM [95% CI: 418.3–824.7] for PAF and 405.94 ± 135.83 µM [95% CI: 275.2–536.7] for ADP-induced aggregation. In contrast, Paracetamol showed significantly weaker inhibition, with markedly higher IC_50_ values of 121,042.32 ± 73,625.26 µM [95% CI: 52,537.9–189,546.7] (PAF) and 50,720.23 ± 17,357.19 µM [95% CI: 34,694.5–66,745.9] (ADP), indicating low antiplatelet potency (Figure 3).

I. Paracetamol

Several studies have investigated the effects of paracetamol on platelet function, focusing primarily on activation pathways mediated by ADP. According to Driver et al., paracetamol exerts minimal effects on platelet aggregation at therapeutic doses. However, intravenous administration at higher doses can lead to a mild, transient inhibition of platelet function, primarily through reduced thromboxane A_2_ synthesis, although the clinical significance of this effect is considered low [22].

Similarly, Hinz et al. (2007) demonstrated that paracetamol acts predominantly as a selective COX-2 inhibitor, with only moderate inhibition of COX-1 (approximately 56% at peak plasma concentration). Since significant inhibition of platelet aggregation typically requires greater than 95% COX-1 inhibition, paracetamol does not substantially impair platelet function, supporting its favorable safety profile compared to traditional NSAIDs [146]. Further evidence from Graham et al. (2001) indicated that paracetamol inhibits prostaglandin production in intact human cells by modulating the cPLA_2_-α/COX-2 pathway. Although this study primarily addressed prostaglandin suppression, it also noted that paracetamol exhibits weak antiplatelet effects, consistent with its limited capacity to inhibit platelet aggregation compared to classical NSAIDs [147].

Additionally, a systematic review by Kao et al. (2022) evaluated the impact of acetaminophen on platelet function and concluded that acetaminophen has no significant effect on platelet aggregation or platelet count, deeming it safe to continue during therapies requiring intact platelet function, such as platelet-rich plasma injections [148]. Clinical data from Gonzalez-Valcarcel et al. (2016) further support these findings. In a large cohort of patients with recent ischemic stroke, paracetamol use was not associated with an increased risk of major bleeding events, unlike ibuprofen, suggesting minimal impact on platelet function [149]. Finally, Catella-Lawson et al. (2001) investigated the interactions between cyclooxygenase inhibitors and aspirin, concluding that paracetamol does not significantly interfere with aspirin’s antiplatelet action and, by itself, has only minimal effects on platelet function [150].

In our experimental setup, paracetamol was initially prepared at a concentration of 500 mg in 3 mL of BSA solution, corresponding to an extremely high starting concentration relative to typical physiological plasma levels. As a result, the measured IC_50_ for inhibition of ADP-induced platelet aggregation was approximately 50.7 mM (50,720 μM) (Figure 5), substantially higher than values reported in the literature. For instance, in the study by Munsterhjelm et al. (2005), paracetamol was added ex vivo to platelet-rich plasma (PRP) from healthy donors at concentrations ranging from 10 to 80 μg/mL. They observed a concentration-dependent inhibition of ADP-induced aggregation, with an estimated IC_50_ between 15 and 30 μg/mL (approximately 100–200 μM). These concentrations fall within the plasma range typically achieved following oral doses of 500–1000 mg (10–20 μg/mL), suggesting that even standard therapeutic dosing may moderately inhibit platelet function via the ADP pathway [38].

The large discrepancy between our measured IC_50_ and literature values is likely attributable to the supraphysiological concentration of paracetamol used in our assay, which may have led to non-specific inhibitory effects, off-target interactions, or even membrane disruption, thereby artificially inflating the IC_50_ value. It is well understood that excessively high drug concentrations can distort pharmacodynamic outcomes, especially in in vitro models. Future experiments should aim to use starting concentrations that better reflect therapeutic plasma levels, to yield more physiologically relevant and directly comparable results.

Although paracetamol is not clinically indicated as an antiplatelet agent, our findings suggest it may exert a weak, dose-dependent inhibitory effect on platelet aggregation, particularly through the ADP pathway. While the supraphysiological concentrations used in our in vitro model likely overstate this effect, the result raises the possibility of pharmacodynamic interactions in polypharmacy contexts, especially in patients receiving antiplatelet therapy. However, the clinical relevance of this observation remains uncertain and would require targeted pharmacokinetic studies under therapeutic conditions.

II. Thiocolchicoside

In parallel, the platelet effects of thiocolchicoside were investigated. Historically, the study by Brossi et al. (1987) reported very low inhibitory activity of thiocolchicoside on platelet aggregation in vitro, in contrast to its derivative thiocolchicine, which exhibited potent inhibition comparable to colchicine [151]. That study did not specify the agonist used, and no IC_50_ values were provided. More recent literature has largely supported the view that thiocolchicoside does not significantly impact platelet function, with minimal or no inhibition reported against ADP, PAF, or other platelet activation pathways [152].

However, in our experimental study, thiocolchicoside demonstrated notable inhibitory activity in a dose-dependent manner, with IC_50_ values of 621.5 μM for PAF-induced aggregation and 405.9 μM for ADP-induced aggregation, indicating slightly greater potency against the ADP pathway (Figure 5). Although these values are relatively high in absolute terms, they are substantially lower than the IC_50_ values obtained for clopidogrel under the same conditions (3291.07 μM for ADP and 281.01 μM for PAF), which was used as a positive control. This comparison is especially relevant, as clopidogrel is a well-established antiplatelet agent, and its elevated IC_50_ values in our study reflect the absence of metabolic activation and other in vivo pharmacokinetic processes.

The standard deviations observed (201.86 μM for PAF and 135.83 μM for ADP) suggest some variability, yet the results remain consistently stronger than expected based on the existing literature. Taken together, our findings challenge the prevailing view that thiocolchicoside lacks antiplatelet activity and suggest that under certain experimental conditions, the compound may exhibit moderate inhibitory effects, particularly when benchmarked against clopidogrel in vitro. These observations warrant further investigation into the potential off-target or previously unrecognized effects of thiocolchicoside on platelet function.

Among analgesic compounds, thiocolchicoside demonstrated moderate inhibitory activity against both PAF- and ADP-induced platelet aggregation, with IC_50_ values in the low micromolar range, indicating unexpected antiplatelet potency under in vitro conditions. In contrast, paracetamol exhibited weak inhibition, with IC_50_ values exceeding 50 mM, likely reflecting its well-established safety profile and limited COX-1 inhibition. Notably, the IC_50_ values for thiocolchicoside were lower than those of clopidogrel under the same assay conditions—an intriguing finding, given the absence of prior evidence supporting its antiplatelet activity. While thiocolchicoside’s mechanism remains unclear, these results suggest potential off-target effects or experimental artifacts that merit further investigation. Collectively, these data reveal heterogeneous antiplatelet properties among analgesics, with implications for drug–drug interactions in patients receiving concurrent antithrombotic therapy.

##### β-blockers—Atenolol, Propranolol, Metoprolol, and Sotalol

The effect of β-blockers on platelet function varies notably based on their receptor selectivity and lipophilicity. Non-selective, lipophilic agents like propranolol (non-selective β_1_/β_2_-blocker), timolol (non-selective β_1_/β_2_-blocker), and pindolol (non-selective β_1_/β_2_-blocker with partial agonist activity) exhibit stronger antiplatelet effects than β_1_-selective blockers such as atenolol, metoprolol, and bisoprolol (all β_1_-selective blockers) [153,154].

Mechanistically, β_2_-adrenoceptors mediate platelet inhibition. In human platelets, β_2_-agonists like isoprenaline significantly inhibit aggregation induced by various excitatory agonists (ADP, collagen, thrombin, adrenaline) through the elevation of cyclic AMP levels, while β_1_-agonists do not have this effect. Rat platelets also express β_2_-adrenoceptors, though the inhibitory response is less prominent compared to human platelets [155]. This suggests that β_2_-receptor activity is critical for the antiplatelet properties observed with non-selective β-blockers.

Although the study by Sharma et al. (2013) did not directly evaluate the antiplatelet or anti-inflammatory properties of β-blockers, it offers valuable insight into prescribing patterns in stable coronary heart disease, showing that β-blockers such as atenolol, metoprolol, and carvedilol were commonly used, with notable variation across primary, secondary, and tertiary healthcare levels in India [156].

Within the present study, interestingly, some β-blockers showed potent anti-ADP and anti-PAF effects (Figure 4). For example, Propranolol exhibited the strongest inhibitory activity, with the lowest IC_50_ values of 346.66 ± 129.19 µM [95% CI: 233.1–460.2] for PAF and 161.86 ± 90.22 µM [95% CI: 85.7–238.1] for ADP. In contrast, Sotalol showed the weakest inhibition, with the highest IC_50_ values of 9901.80 ± 4750.39 µM [95% CI: 5879.6–13,923.9] (PAF) and 10,661.67 ± 3755.12 µM [95% CI: 7027.8–14,295.5] (ADP). Atenolol demonstrated moderate effects with IC_50_ values of 6284.80 ± 3008.44 µM [95% CI: 3721.1–8848.5] (PAF) and 5560.11 ± 3082.81 µM [95% CI: 2938.1–8182.1] (ADP), while Metoprolol showed slightly stronger activity, with IC_50_ values of 2361.72 ± 360.88 µM [95% CI: 1995.3–2728.2] and 1287.08 ± 472.37 µM [95% CI: 906.3–1667.9], respectively.

I. Atenolol

Although widely prescribed for its cardiovascular benefits, atenolol, a β_1_-selective adrenergic blocker, appears to have limited direct antiplatelet activity. In preclinical studies, atenolol slightly potentiated stimulated platelet aggregation and arachidonic acid release in rat models but had no measurable effect on thromboxane B_2_ (TxB_2_) production [157]. Interestingly, while it reduced platelet adhesion, its effect on aggregation remained minimal, suggesting an incomplete inhibitory profile. Clinically, a randomized crossover trial by Punda et al. (2005) demonstrated that atenolol failed to significantly reduce platelet aggregation in hypertensive patients, while propranolol showed a clear inhibitory effect, highlighting a pharmacodynamic difference between β_1_-selective and non-selective β-blockers [153].

Our findings are consistent with these observations. In the present study, atenolol exhibited very weak inhibition of platelet aggregation in human platelet-rich plasma (hPRP), with IC_50_ values of 6284.8 μM for PAF-induced aggregation and 5560.1 μM for ADP-induced aggregation (Figure 4). These results point to minimal antiplatelet potency, especially when compared to agents like clopidogrel, which achieved near-complete inhibition at sub-micromolar concentrations. Additionally, in a comparative animal model, Iwamura et al. (1983) reported an IC_50_ of approximately 3600 μM for atenolol in guinea pig PRP stimulated with ADP—again markedly weaker than the response observed with propranolol [158].

Despite its prevalence in clinical practice, as shown in an Indian prescribing audit where 37.8% of patients with stable coronary artery disease on β-blockers were prescribed atenolol [156], these cumulative data underscore atenolol’s lack of direct platelet-inhibitory effects. This is likely due to its selectivity for β_1_-adrenergic receptors, which are primarily cardiac and not significantly expressed on platelets, and its absence of membrane-stabilizing or calcium-channel modulating actions, both of which contribute to the antiplatelet activity of more pharmacodynamically diverse β-blockers such as propranolol.

II. Propranolol

Propranolol, a non-selective β-blocker, has consistently demonstrated broad and potent antiplatelet effects across preclinical, in vitro, and clinical studies. Unlike β_1_-selective agents, propranolol not only attenuates platelet aggregation but also modulates key biochemical pathways involved in platelet activation. In animal studies, propranolol was shown to dose-dependently inhibit platelet aggregation, reduce arachidonic acid release, and suppress thromboxane B_2_ (TxB_2_) production, suggesting involvement in prostaglandin synthesis modulation [159].

These effects have been confirmed in human models. At therapeutic concentrations, propranolol inhibited collagen- and thrombin-induced aggregation, reduced TxB_2_ levels in both PRP and whole blood, and lowered β-thromboglobulin (B-TG) release, while also suppressing spontaneous clot formation, indicating a broad and physiologically relevant inhibition of platelet function and thromboxane A_2_ generation [160]. These mechanisms may underlie propranolol’s cardioprotective effects in conditions like coronary artery disease, where platelet hyperactivity plays a key role. These mechanisms may underlie propranolol’s cardioprotective effects in conditions like coronary artery disease, where platelet hyperactivity plays a key role.

Further, propranolol also increased the aggregation threshold in response to ADP, collagen, and epinephrine, with effects that appear independent of β-receptor blockade, suggesting a direct membrane action, possibly through modulation of calcium availability [161]. In hypertensive patients, propranolol significantly lowered the threshold for irreversible ADP- and adrenaline-induced aggregation compared to metoprolol (β_1_-selective blocker) and reduced basal platelet cAMP, reinforcing its stronger effect on platelet activation suppression [162].

Our experimental findings align with these reports. In the present study, propranolol exhibited pronounced antiplatelet activity, with IC_50_ values of 346.7 μM for PAF-induced aggregation and 161.9 μM for ADP-induced aggregation in human platelet-rich plasma (hPRP), indicating a pronounced inhibitory effect, particularly in the ADP pathway (Figure 4). Notably, clopidogrel was used as a positive control, providing a benchmark for effective antiplatelet activity, against which propranolol showed moderate but still significant and dose-dependent activity.

These findings align with previous reports. Weksler et al. (1977) observed that propranolol at 20 μM inhibited ADP-induced aggregation by ~50% in hPRP [161]. Iwamura et al. (1983) showed propranolol inhibited ADP-induced aggregation in guinea pig PRP with an IC_50_ of ~210 μM, confirming cross-species efficacy [158]. In a detailed in vitro analysis, Anfossi et al. (1989) reported that propranolol at 10^−4^ M (100 μM) inhibited ADP-induced aggregation by 55% and reduced thromboxane B_2_ production by up to 45% in PRP, further supporting a dual mechanism involving both platelet signaling and eicosanoid modulation [160].

In a clinical setting, Punda et al. (2005) demonstrated that oral propranolol significantly reduced ADP- and collagen-induced platelet aggregation in patients with moderate essential hypertension, with a mean decrease of 19% in ADP-induced aggregation, while atenolol produced no statistically significant effect [153]. Notably, while propranolol showed stronger inhibition in the ADP pathway compared to the PAF pathway, the literature provides very limited data regarding the role of β-blockers on PAF-induced platelet aggregation, which may explain the comparatively higher IC_50_ values observed in our PAF experiments. Taken together, these results support the view that propranolol’s antiplatelet effects are both dose-dependent and mechanistically distinct, likely involving membrane-stabilizing and calcium-modulating activity in addition to β-adrenergic blockade. These findings reinforce the potential of propranolol as a dual-action cardiovascular agent, providing hemodynamic and antithrombotic benefits.

III. Metoprolol

Among newer β-blockers, nebivolol—a β_1_-selective agent with nitric oxide–mediated vasodilatory properties—has demonstrated greater efficacy in reducing ADP-induced platelet aggregation. In patients receiving dual antiplatelet therapy, nebivolol produced a more pronounced inhibitory effect compared to bisoprolol, while metoprolol and carvedilol showed no significant differences, suggesting that nebivolol may be better suited to complement antiplatelet strategies [163].

In our study, we evaluated the antiplatelet potential of metoprolol, a widely used β_1_-selective adrenergic blocker. The drug was prepared at a concentration of 100 mg in 3 mL of BSA and tested in human platelet-rich plasma (hPRP). Metoprolol demonstrated weak inhibitory activity, with IC_50_ values of 2361.7 μM for PAF-induced aggregation and 1287.1 μM for ADP-induced aggregation, indicating that very high concentrations were needed to produce moderate inhibition (Figure 4). In contrast, the positive control, clopidogrel, produced near-complete inhibition at sub-micromolar concentrations, highlighting the limited potency of metoprolol in modulating platelet function.

These results are consistent with clinical and experimental evidence. Winther et al. (1986) reported that metoprolol at 100–200 mg/day did not significantly reduce ADP-induced platelet aggregation or alter platelet cAMP levels in hypertensive patients [162]. A broader evaluation by Bonten et al. (2014) showed that β_1_-selective blockers had no statistically significant effect on platelet aggregation (SMD = 0.03, 95% CI: −0.12 to 0.19) [154]. Additionally, metoprolol at 200 mg/day failed to reduce platelet aggregability under stress conditions in healthy volunteers, further confirming its limited pharmacologic influence on platelet activation [164].

Altogether, these findings suggest that metoprolol’s lack of membrane-stabilizing, calcium channel-blocking, or nitric oxide–mediated activity contributes to its poor performance in platelet inhibition, distinguishing it from more effective agents like propranolol or nebivolol. As such, its cardioprotective benefit is likely confined to hemodynamic modulation rather than direct antiplatelet effects.

IV. Sotalol

Among β-blockers, sotalol is unique for its combination of non-selective β-adrenergic antagonism and Class III antiarrhythmic action, yet its role in modulating platelet function appears limited and inconsistent. Mechanistically, sotalol’s primary effect lies in potassium channel blockade, explaining its antiarrhythmic action but not contributing significantly to platelet inhibition. While mild in vitro effects have been noted, Korbut et al. (1998) demonstrated that sotalol’s antithrombotic activity is likely indirect, mediated by enhanced endothelial release of tissue plasminogen activator (t-PA) via prostacyclin and nitric oxide pathways [165]. Supporting this, Premuzic Mestrovic et al. (2023) reported that high-dose sotalol induced occlusion-like syndromes with widespread thrombosis in rats, which were reversed by BPC 157, indicating a vascular—not platelet—mechanism underlying its thrombotic profile [166].

These findings align with our current study, in which sotalol exhibited the weakest antiplatelet activity among the β-blockers tested. We observed IC_50_ values of 9901.8 μM for PAF-induced aggregation and 10,661.7 μM for ADP-induced aggregation in human platelet-rich plasma (hPRP), indicating extremely low potency. These results are consistent with earlier pharmacological reports that described sotalol’s inhibition as weak and inconsistent, with IC_50_ values estimated between 500 and 1000 μM, and highlighted its lack of efficacy at therapeutic concentrations [167].

Altogether, both our data and existing literature indicate that sotalol has negligible antiplatelet activity, particularly when compared to more potent non-selective β-blockers like propranolol. Its potential antithrombotic effects, if any, appear to be mediated through indirect vascular mechanisms rather than direct platelet inhibition, limiting its relevance in clinical settings where platelet hyperactivity is a central concern. Taken together, these results suggest that non-selective β-blockers like propranolol possess superior antiplatelet effects compared to β_1_-selective blockers, likely due to additional membrane-stabilizing or calcium-channel modulating properties independent of β-adrenergic receptor antagonism.

The β-blocker class demonstrated marked variability in antiplatelet potency, largely influenced by receptor selectivity and intrinsic pharmacologic properties. Propranolol, a non-selective and lipophilic β-blocker, showed the strongest inhibitory activity across both PAF and ADP pathways, with IC_50_ values well below those of the other agents tested, likely due to additional membrane-stabilizing and calcium-modulating effects. Metoprolol and Atenolol, both β_1_-selective agents, exhibited moderate to weak antiplatelet activity, while Sotalol, despite its β-blocking and Class III antiarrhythmic actions, showed minimal direct platelet inhibition, consistent with its known endothelial- rather than platelet-based effects. Overall, non-selective β-blockers exhibited superior potency, supporting mechanistic links between β_2_-receptor blockade and platelet modulation.

##### Other Antihypertensives—Candesartan and Valsartan

With respect to the other classic antihypertensive drugs assessed, Candesartan exhibited significantly lower IC_50_ values compared to Valsartan, indicating a stronger inhibitory effect on platelet aggregation (Figure 5). Specifically, Candesartan showed IC_50_ values of 3907.52 ± 1299.50 µM [95% CI: 1382.6–6432.4] for PAF and 3895.13 ± 2045.13 µM [95% CI: 1963.6–5826.7] for ADP-induced aggregation. In contrast, Valsartan presented notably higher IC_50_ values of 13,572.57 ± 5513.50 µM [95% CI: 4550.9–22,594.2] (PAF) and 11,172.65 ± 5076.32 µM [95% CI: 3690.6–18,654.7] (ADP), suggesting substantially weaker antiplatelet activity. These findings indicate that Candesartan may have greater potential to modulate platelet function than Valsartan.

I. Candesartan

Several studies have explored the effects of candesartan and valsartan on platelet function. In direct comparisons, Sato et al. found that hypertensive patients treated with candesartan showed higher platelet aggregation responses to ADP and thromboxane A_2_ stimulation compared to those treated with losartan, indicating a weaker antiplatelet effect of candesartan [167]. Fogari et al. also demonstrated that losartan more effectively reduced thrombin-mediated platelet aggregation and enhanced fibrinolysis compared to candesartan [168].

Multiple studies evaluated candesartan’s effects independently. Specifically, Núñez et al. reported that candesartan failed to inhibit thromboxane A_2_-dependent platelet activation in vitro [169]. Jiménez et al. showed that candesartan did not significantly reduce platelet activation or P-selectin expression in stroke-prone hypertensive rats [170]. while Nossaman et al. confirmed that candesartan did not inhibit arachidonic acid-induced platelet aggregation [171]. Furthermore, Ishikawa et al. demonstrated that candesartan significantly inhibited platelet-leukocyte-endothelial interactions by reducing oxidative stress and P-selectin-mediated adhesion, suggesting an indirect antithrombotic effect [172]. Fogari et al. observed that candesartan improved fibrinolytic parameters but did not significantly reduce platelet aggregation in diabetic hypertensive patients [168]. Also, Hallevi et al. found that candesartan reduced neutrophil adhesion to endothelial cells, indirectly suggesting a vascular protective role [173]. Moreover, Buda et al. showed candesartan lowered vascular inflammatory markers such as pentraxin-3 (PTX3), which may contribute to reduced thrombotic risk [174], and Al-Azzam et al. confirmed that candesartan did not interfere with aspirin’s antiplatelet activity [175].

Similarly, candesartan exhibited high IC_50_ values in our PRP assays: 3907.5 μM for PAF-induced aggregation and 3895.1 μM for ADP-induced aggregation. Although candesartan appeared more potent than valsartan under the same conditions, its IC_50_ values still suggest only weak inhibition of platelet aggregation at high concentrations. Like valsartan, candesartan is known to exhibit strong albumin binding, with >99% of the drug bound to plasma proteins, predominantly albumin [176,177]. This high affinity likely contributes to reduced availability of the unbound, pharmacologically active fraction in vitro. The presence of bovine serum albumin (BSA) in our experimental system may have amplified drug sequestration, as BSA is known to mimic human serum albumin in its binding properties and can significantly reduce the effective free concentration of highly protein-bound drugs like ARBs [178,179]. Furthermore, no prior studies have provided definitive IC_50_ values for candesartan in the context of PAF- or ADP-induced platelet aggregation, making our findings among the first to quantify its inhibitory potential under controlled in vitro conditions. It is also important to consider that high experimental concentrations may result in non-specific interactions, saturation effects, or alterations in solubility, all of which can influence IC_50_ interpretation. These results highlight the importance of accounting for protein-binding dynamics in pharmacological assays and call for further research to establish standardized testing conditions for angiotensin receptor blockers (ARBs) like candesartan.

II. Valsartan

Valsartan has shown more consistent antiplatelet effects across studies. To be exact, Núñez et al. observed that valsartan moderately reduced thromboxane A_2_-induced platelet activation only at high concentrations [169], while Jiménez et al. found valsartan did not significantly reduce platelet activation markers in hypertensive rats [170]. Experimental studies showed valsartan and its metabolite inhibited collagen-induced platelet aggregation at therapeutic concentrations [179]. In Serebruany et al.’s study it was demonstrated that valsartan and V4HV inhibited ADP- and collagen-induced platelet aggregation in vitro, with V4HV being more potent [180]. Furthermore, the VIP trial confirmed that valsartan reduced platelet aggregation and activation markers in hypertensive diabetic patients [181]. Moreover, Wu et al. reported valsartan reduced COX-2 and TXB2 levels and inhibited platelet aggregation through the p38MAPK and NF-κB pathways [182], and in the AVID trial, valsartan combined with aliskiren significantly inhibited ADP-induced platelet aggregation and platelet activation markers even on top of low-dose aspirin [183].

Kalinowski et al. suggested that valsartan’s antiplatelet effects were mediated by nitric oxide (NO) release from platelets [184], while López-Farré et al. confirmed valsartan reduced platelet activation markers independently of blood pressure reduction [185]. In Sironi et al.’s study it was shown that valsartan inhibited angiotensin II–induced PAI-1 secretion in vascular smooth muscle cells with an IC_50_ of 21 nM, indicating a beneficial effect on fibrinolytic balance [186]. Furthermore, Liu et al. found that valsartan attenuated postprandial increases in soluble P-selectin after a high-fat meal, suggesting an indirect antiplatelet effect [187]. Lastly, Al-Azzam et al. confirmed valsartan did not interfere with aspirin’s antiplatelet effects [175], and Malinin et al. highlighted valsartan’s potential antiplatelet activity beyond conventional antiplatelet drugs [188].

In our platelet-rich plasma (PRP) assays, valsartan demonstrated high IC_50_ values of 13,572.5 μM for PAF-induced aggregation and 11,172.6 μM for ADP-induced aggregation, indicating weak antiplatelet activity. These IC_50_ values are notably higher than those expected for a pharmacologically active agent and likely reflect specific experimental factors. One key consideration is valsartan’s very high plasma protein binding (~96%), primarily to albumin [189]. Notably, valsartan has been shown to interact significantly with oxidized albumin, suggesting a high affinity for BSA [190]. Since BSA was used as the solubilizing medium in our experiments, it is plausible that a substantial proportion of the drug was sequestered in drug–protein complexes, thereby reducing the free (active) concentration of valsartan available to interact with platelets. Additionally, the use of high drug concentrations in our dose-response curves may have led to saturation of albumin binding sites, complicating the pharmacodynamic assessment further [191]. These factors collectively explain the elevated IC_50_ values observed in our study. As specific IC_50_ data for valsartan’s direct effects on platelet aggregation (particularly in response to ADP or PAF) are currently lacking in the published literature, our findings underscore the need for standardized in vitro platelet function studies with careful control of protein-binding conditions.

Our findings indicate that Candesartan exhibits greater inhibitory potency than Valsartan against both PAF- and ADP-induced platelet aggregation under identical in vitro conditions. While both agents showed relatively high IC_50_ values—suggesting weak intrinsic antiplatelet activity—the data point to class variability within angiotensin receptor blockers (ARBs). Despite literature describing indirect anti-inflammatory and vascular effects of both agents, the observed differences may stem from differential protein-binding profiles, receptor affinities, or off-target interactions. These results reinforce the need for standardized in vitro models that account for protein-binding dynamics and highlight Candesartan’s potential for broader modulatory effects on platelet function, particularly in comparison to Valsartan.

The comparative IC_50_ profiling across both the ADP and PAF pathways presents a novel mechanistic layer to our understanding of drug-specific antiplatelet effects. Prior studies have largely focused on either classical ADP or COX-related mechanisms. However, PAF-mediated platelet activation remains under-characterized despite its known involvement in thrombo-inflammatory diseases. This study addresses that gap and builds on recent calls in the literature—such as Tsoupras et al. (2024) [192]—for more systematic investigations of the PAF pathway using human PRP models. It is important to note that these results are based on platelets from healthy donors, and responses may differ in patients with cardiovascular or inflammatory conditions.

To facilitate clearer comparison of antiplatelet potency across all compounds tested, Table 1 summarizes IC_50_ values, standard deviations, and 95% confidence intervals for each drug against both PAF- and ADP-induced platelet aggregation. In addition, mean percentage aggregation values (±SD) for each drug under both pathways are included to provide a more comprehensive picture of platelet response. The table ranks the drugs from most to least potent in each pathway, highlighting class-specific patterns of selectivity and overall inhibitory strength.

### 3.3. Evaluation of Synergistic Interactions on Platelet Reactivity of Drug Combinations (1:1 Ratio)

Statistical analysis of drug efficacy was performed using the PSPP software suite. Initially, a one-way ANOVA was conducted to assess whether significant differences existed among the various treatment groups. Descriptive statistics and confidence intervals were generated to evaluate variability within and between treatments. Following the ANOVA, independent-samples *t*-tests were applied to compare the IC_50_ values of each single-agent drug to those of its respective combinations. Each test focused on combinations that included the reference drug, allowing for the determination of whether observed differences were statistically significant (*p* < 0.05). These comparisons were repeated across all five primary drugs (clopidogrel, lornoxicam, propranolol, paracetamol, and candesartan) using the 11 predefined therapeutic combinations.

I. Inhibition Potency of Clopidogrel vs. Multi-Drug Combinations

**Figure 6:**
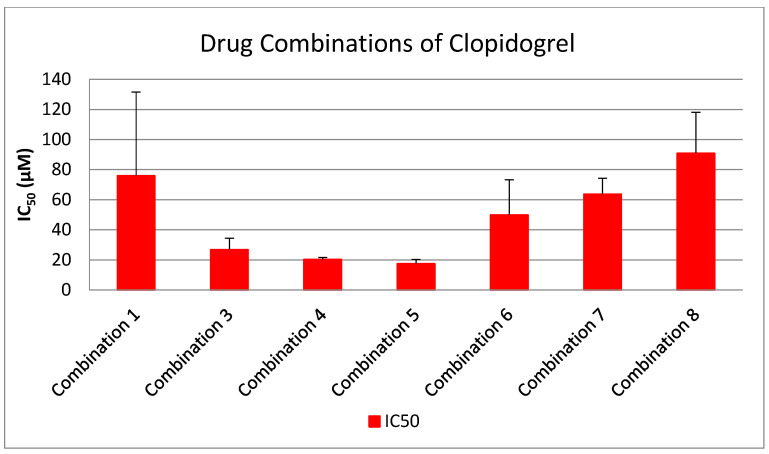
Bar graph showing IC_50_ values (μM ± SD) for clopidogrel and various drug combinations in inhibiting PAF-induced platelet aggregation. Combinations 1–8 showed statistically significant reductions in IC_50_ compared to clopidogrel (*p* < 0.05), while combinations 9 and 10 did not demonstrate a statistically significant difference (*p* > 0.05). Each combination was tested in a 1:1 ratio. Data represent mean ± standard deviation from 3 to 6 independent experiments per drug.

As shown in Table 1, clopidogrel alone exhibited an IC_50_ of 281.01 μM (±176.03), while as illustrated in Figure 6, all combinations tested except combinations 9 (Propranolol + Clopidogrel) and 10 (Propranolol + Clopidogrel + Candesartan) resulted in statistically significant reductions in IC_50_ (*p* < 0.05). The combination of all five agents—lornoxicam, clopidogrel, paracetamol, propranolol, and candesartan—demonstrated the lowest IC_50_ (20.26 ± 1.40 μM), indicating enhanced potency through synergistic effects. Combinations 9 and 10 did not differ significantly from clopidogrel alone (*p* = 0.105 and *p* = 0.147, respectively), suggesting a limited additive effect from propranolol or candesartan in these specific pairings.

II. Inhibition Potency of Lornoxicam vs. Multi-Drug Combinations

**Figure 7:**
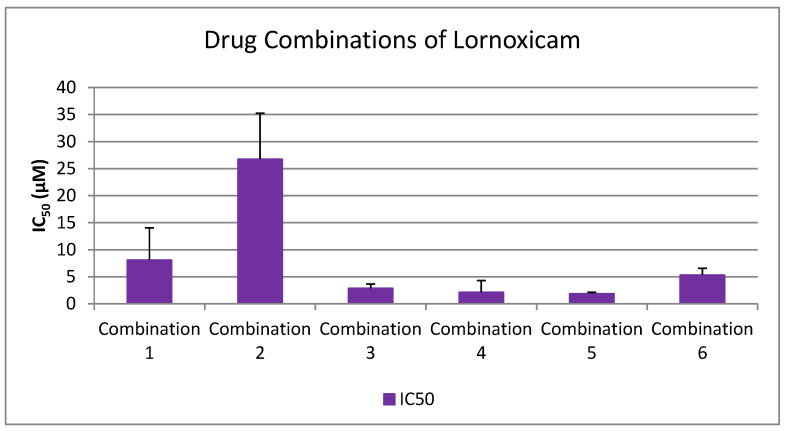
Bar graph showing IC_50_ values (μM ± SD) for lornoxicam and its statistically significant combinations in inhibiting PAF-induced platelet aggregation. All combinations tested (1–6) resulted in significantly lower IC_50_ values compared to lornoxicam alone (*p* < 0.001), indicating potent synergistic effects. Each combination was administered in a 1:1 molar ratio. Data represent mean ± standard deviation from 3 to 6 independent experiments.

As shown in Table lornoxicam alone displayed an IC_50_ value of 313.99 ± 58.34 μM in PAF-induced platelet aggregation. However, as shown in Figure 7, when combined with other agents, including clopidogrel, paracetamol, candesartan, and propranolol, in a 1:1 equimolar ratio, the resulting IC_50_ values were significantly reduced (*p* < 0.001 for all combinations). The most pronounced synergistic effect was observed in the full five-drug combination (Combination 4), which achieved an IC_50_ as low as 2.16 ± 0.15 μM. These findings support the hypothesis that lornoxicam’s antiplatelet activity can be greatly enhanced when used in multi-drug regimens, particularly in combinations targeting complementary pathways of platelet activation.

III. Inhibition Potency of Propranolol vs. Multi-Drug Combinations

**Figure 8:**
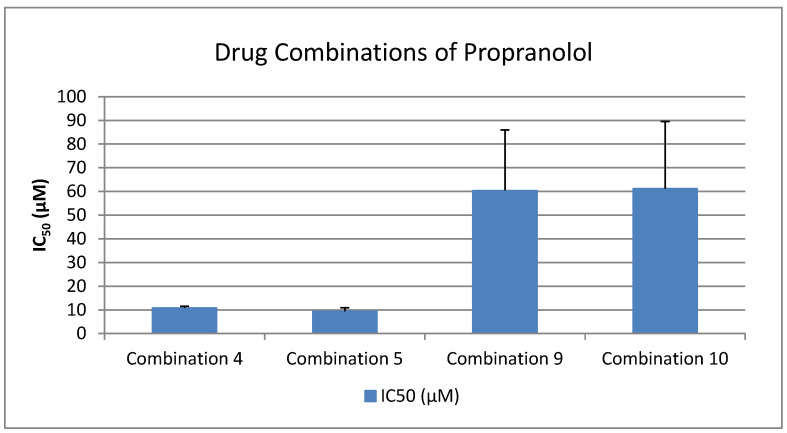
Bar graph showing IC_50_ values (μM ± SD) for propranolol and its statistically evaluated combinations in inhibiting PAF-induced platelet aggregation. Combinations 4, 5, 9, and 10 showed significantly lower IC_50_ values compared to propranolol alone (*p* < 0.05), indicating synergistic enhancement. Combination 11, however, did not produce a statistically significant difference (*p* = 0.050). All combinations were tested in a 1:1 molar ratio. Data represent mean ± standard deviation from 3 to 6 independent experiments.

As shown in Table 1, propranolol alone demonstrated an IC_50_ of 346.66 ± 129.19 μM against PAF-induced platelet aggregation. Nevertheless, as shown in Figure 8, when combined in equimolar 1:1 ratios with other agents such as lornoxicam, clopidogrel, or candesartan, significant reductions in IC_50_ were observed for Combinations 4, 5, 9, and 10 (*p* < 0.05), confirming synergistic inhibition of platelet function. In contrast, Combination 11 (propranolol + candesartan) did not show a statistically significant difference from propranolol alone (*p* = 0.050), suggesting that this pairing may not enhance antiplatelet efficacy. These findings further support the benefit of multi-agent regimens targeting complementary platelet activation pathways.

IV. Inhibition Potency of Paracetamol vs. Multi-Drug Combinations

**Figure 9:**
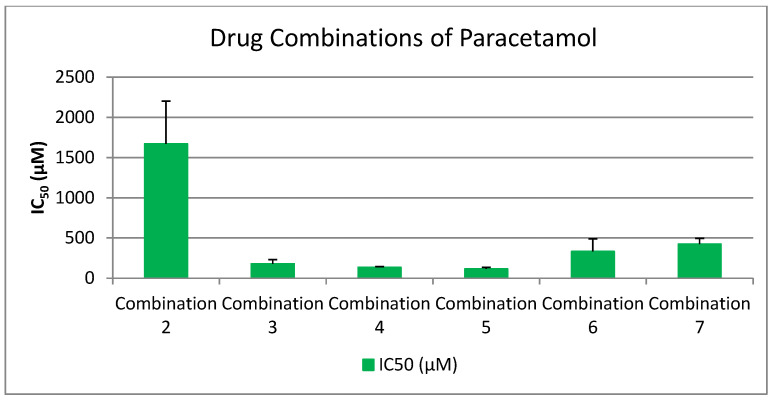
Bar graph showing IC_50_ values (μM ± SD) for paracetamol and its statistically evaluated combinations in inhibiting PAF-induced platelet aggregation. All tested combinations (2–7) demonstrated significantly lower IC_50_ values compared to paracetamol alone (*p* < 0.05), indicating synergistic inhibition. Each combination was administered in a 1:1 molar ratio. Data represent mean ± standard deviation from 3 to 6 independent experiments.

As shown in Table 1, paracetamol alone exhibited a markedly high IC_50_ value of 121,042.3 ± 73,625.26 μM, reflecting limited antiplatelet potency against the PAF pathway. However, as illustrated in Figure 9, when co-administered in an equimolar 1:1 combination with drugs such as lornoxicam, clopidogrel, propranolol, or candesartan (Combinations 2–7), a significant reduction in IC_50_ was consistently observed (*p* < 0.05 for all comparisons). The most potent synergistic effect occurred in Combination 4 (paracetamol + lornoxicam + clopidogrel + propranolol + candesartan), which reduced the IC_50_ to 135.08 ± 9.34 μM. These results highlight the potential of paracetamol to enhance antiplatelet responses when used in rational multi-drug regimens, despite its minimal activity as a standalone agent.

V. Inhibition Potency of Candesartan vs. Multi-Drug Combinations

**Figure 10:**
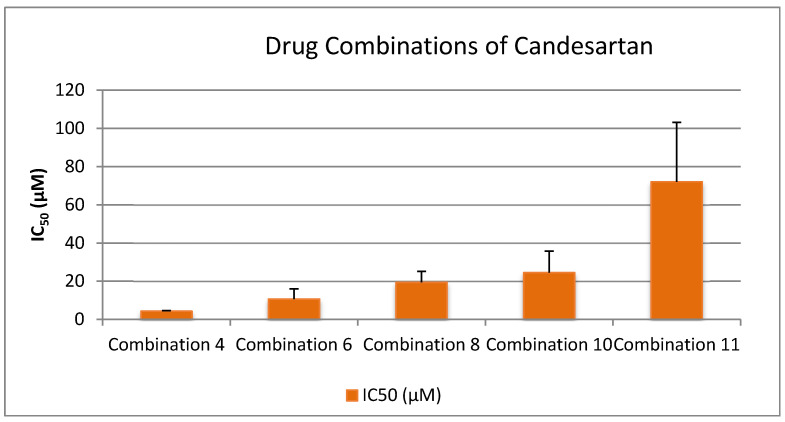
Bar graph showing IC_50_ values (μM ± SD) for candesartan and its statistically evaluated combinations in inhibiting PAF-induced platelet aggregation. All tested combinations (4, 6, 8, 10, 11) resulted in significantly lower IC_50_ values compared to candesartan alone (*p* < 0.05), indicating strong synergistic effects. Each combination was tested at a 1:1 molar ratio. Data represent mean ± standard deviation from 3 to 5 independent experiments.

As shown in Table 1, candesartan alone displayed a high IC_50_ value of 3907.52 ± 1299.50 μM in PAF-induced platelet aggregation, suggesting minimal efficacy as a monotherapy in this pathway. In contrast, as illustrated in Figure 10, when combined in equimolar ratios with agents such as lornoxicam, clopidogrel, paracetamol, or propranolol (Combinations 4, 6, 8, 10, and 11), all combinations demonstrated statistically significant reductions in IC_50_ (*p* < 0.05). The greatest synergistic response was observed in Combination 4 (all five agents), which achieved an IC_50_ of 4.32 ± 0.30 μM. These findings suggest that candesartan, though weak alone, can contribute meaningfully to platelet inhibition when integrated into targeted multi-drug combinations affecting the PAF pathway.

Across all five agents tested, combination therapies consistently demonstrated significantly lower IC_50_ values compared to their corresponding single-drug controls, indicating enhanced inhibition of PAF-induced platelet aggregation via synergistic mechanisms. Clopidogrel showed the most robust synergistic effect when co-administered with lornoxicam, paracetamol, and candesartan, particularly in the five-drug Combination 4 (IC_50_: 20.26 ± 1.40 μM), while combinations 9 and 10 did not differ significantly. Lornoxicam alone showed moderate inhibition, but its efficacy was dramatically enhanced when included in any of the tested combinations (1–6), all showing *p* < 0.001. Propranolol also benefited significantly from combination therapy, with Combinations 4, 5, 9, and 10 showing marked reductions in IC_50_; only Combination 11 (propranolol + candesartan) failed to reach significance (*p* = 0.050). Paracetamol, which exhibited the highest IC_50_ value as a monotherapy (121,042.3 ± 73,625.26 μM), demonstrated substantial synergistic gains in all combinations tested (2–7), reducing the IC_50_ by several orders of magnitude. Lastly, candesartan—despite its limited potency as a single agent—showed strong synergistic responses in all five combinations tested (4, 6, 8, 10, 11), particularly when part of the full multi-drug regimen. These findings collectively confirm the significant benefit of rational multi-agent strategies in amplifying antiplatelet activity, particularly via the PAF pathway. Thus, this work highlights drug class-specific selectivity and synergy effects, which have not been concurrently reported across such a diverse pharmacological panel. As such, our findings contribute both mechanistic insight and clinical relevance for dual-pathway antiplatelet targeting.

#### Clinical Relevance and Translational Implications

The dual-pathway antiplatelet assessment reveals clinically relevant patterns of drug activity that extend beyond traditional classification. COX-inhibiting NSAIDs such as lornoxicam and diclofenac, while primarily known for anti-inflammatory action, demonstrated potent inhibitory effects on both ADP- and PAF-induced aggregation. This suggests a broader antithrombotic profile that may support their use as adjunctive agents in cardiovascular risk management—particularly in patients with inflammation-driven thrombosis [193]. Although NSAIDs are generally not recommended as frontline antiplatelet therapy due to bleeding risk [194], these findings indicate that selective NSAIDs with favorable IC_50_ profiles could be reconsidered in carefully selected contexts.

Similarly, β-blockers such as propranolol, atenolol, and metoprolol—typically used for rate control and blood pressure management—showed significant anti-ADP activity, potentially offering secondary antiplatelet benefits [154]. The observation that propranolol also induces baseline platelet activation highlights the need for caution and individualized therapy, especially in combination regimens.

Among antihypertensives, ARBs like candesartan and valsartan exhibited balanced but modest inhibition across both pathways. Their role in modulating platelet function may contribute to their cardioprotective effects beyond blood pressure regulation [195]. Analgesics presented the most variability; thiocolchicoside showed robust ADP inhibition, while paracetamol demonstrated weak monotherapy effects but contributed synergistically in combinations—highlighting the complexity of its interaction with platelet function.

These findings may inform rational drug selection and combination strategies for patients at risk of both thrombotic and inflammatory vascular conditions. By profiling pathway-specific inhibitory effects, this study provides mechanistic support for integrating dual-pathway screening into cardiovascular pharmacotherapy development and optimization. These insights underscore the translational importance of characterizing both PAF and ADP interactions when evaluating pharmacological agents with potential cardiovascular applications.

These findings, though based on in vitro human PRP models, may inform future in vivo studies and rational polypharmacy design in clinical settings. Specifically, they suggest potential benefit in targeting multiple platelet activation pathways—PAF and ADP—in high-risk cardiovascular or inflammatory patients. Further clinical investigation is needed to validate these synergistic effects in patient populations and assess bleeding risk versus therapeutic gain.

### 3.4. Results of Toxicological Assessment of Drug-Induced Platelet Aggregation in PRP

Among the drugs tested, only the β-blockers propranolol and atenolol induced measurable platelet aggregation in platelet-rich plasma (PRP) samples in the absence of external agonists, indicating a potential intrinsic pro-aggregatory effect. To further evaluate this phenomenon, both compounds were tested in a dose-dependent manner by sequentially reducing the concentration added to PRP (within a range of 0.1 to 50 mM). Aggregation curves were recorded using light transmission aggregometry, and the peak amplitude of light transmittance was used to quantify platelet activation.

This stepwise protocol revealed clear dose-dependent pro-aggregatory and potentially cytotoxic responses, especially for propranolol, which produced marked aggregation and a loss of optical clarity at higher concentrations—features suggestive of excessive platelet activation or lysis. The IC_50_ range for propranolol was calculated from the responses at 40 μL, 20 μL, and 10 μL, yielding values between 217 μM (0.217 mM) and 476 μM (0.476 mM), while the EC_50_ for the observed platelet aggregation induced by this drug was approximately at 20.38 mM (at least two orders of magnitude higher concentrations than those of the IC_50_ values for this drug against PAF). In contrast, atenolol induced a milder but still measurable pro-aggregatory effect that diminished with decreasing concentration. Its IC_50_, derived from the 100 μL, 80 μL, and 60 μL concentrations, ranged from 3.28 mM to 9.29 mM, while the EC_50_ for the observed platelet aggregation induced by this drug was approximately at 31.77 mM (almost one order of magnitude higher concentrations than those of the IC_50_ values for this drug against PAF).

These data demonstrate that propranolol has a significantly stronger intrinsic effect on platelet aggregation than atenolol, as reflected by both its lower IC_50_ range and lower EC_50_ value. The threshold dose at which no aggregation was observed was lower for atenolol, suggesting a broader safety margin under the tested in vitro conditions.

Overall, these findings underscore that β-blockers—particularly lipophilic agents like propranolol—may elicit direct platelet-activating or cytotoxic effects independent of classical receptor-mediated mechanisms when used in high concentrations. This highlights the importance of functional platelet assays in evaluating off-target pro-thrombotic risks of cardiovascular agents, especially when considering high plasma concentrations or polypharmacy settings.

### 3.5. Results of Desensitization Effects and Intrinsic Platelet Activation

To further investigate the potential intrinsic effects of β-blockers on platelet function, drug-only PRP assays were conducted and aggregation curves were recorded. The results illustrate distinct response patterns following the addition of atenolol and propranolol. In one test involving atenolol, the aggregation curve showed a reduced amplitude, falling within 20–80% of the maximal PAF-induced response. This may reflect a transient inhibitory or desensitizing effect on platelet reactivity. In contrast, the remaining three tests—one with atenolol and both with propranolol—demonstrated significantly enhanced aggregation responses that exceeded the maximal response induced by PAF. These findings suggest that, at high concentrations, β-blockers may directly activate platelets via intrinsic pathways, even in the absence of classical agonists. This interpretation is consistent with clinical data from Winther et al. (1986), who found that propranolol treatment significantly lowered the ADP threshold for irreversible aggregation in hypertensive patients, indicating a pro-aggregatory shift in platelet sensitivity even under therapeutic dosing [162].

This response pattern aligns with earlier observations by Weiss and Rogers (1972), who described abnormal platelet release reactions occurring independently of standard agonist stimulation [196]. Similarly, Trovati et al. (1986) reported that metabolic disturbances such as hypoglycemia can trigger platelet activation through non-traditional mechanisms, supporting the possibility of pharmacologically induced, off-target pro-aggregatory effects [197]. These results underscore the relevance of incorporating drug-only PRP assays into toxicological evaluations. Such assays are crucial for detecting latent platelet-activating properties of drugs, which may not be apparent in conventional agonist-based tests, and could help identify potential thrombotic risks associated with high drug exposure.

### 3.6. Limitations

One important limitation of this study is the exclusive use of platelet-rich plasma (PRP) derived from healthy human donors. This approach was necessary due to ethical considerations, availability, and the controlled nature of our in vitro system. However, platelet responses can vary significantly in patients with cardiovascular disease (CVD), systemic inflammation, or other comorbidities that alter platelet reactivity. Whether the observed inhibitory profiles and synergistic effects apply equally to platelets from clinical populations remains unknown.

Furthermore, the in vitro nature of our experiments, while providing controlled mechanistic screening, lacks direct clinical correlation. The effects observed cannot be extrapolated to therapeutic outcomes in vivo without further pharmacokinetic, safety, or efficacy validation in animal models or human subjects. Future studies should incorporate in vivo models or clinical samples to confirm translational relevance.

Additionally, the use of commercial tablet formulations rather than purified active pharmaceutical ingredients (APIs) represents a methodological limitation. Formulation excipients may influence solubility, bioavailability, or assay interference. Future studies should include standardized APIs to improve reproducibility and comparability.

Lastly, our study focused primarily on IC_50_ quantification, without investigation of the molecular mechanisms underlying the observed platelet inhibition. The absence of mechanistic assays (e.g., receptor-binding studies, COX inhibition, or downstream intracellular signaling analyses) limits the ability to identify specific pharmacological targets. Future mechanistic investigations are warranted to elucidate the exact pathways responsible for drug-induced antiplatelet effects.

## 4. Conclusions

This study provides a comprehensive ex vivo evaluation of the antiplatelet and anti-inflammatory profiles of various drug classes, using dual-pathway assessment of PAF- and ADP-induced platelet aggregation. Clopidogrel served as the positive control, confirming its established ADP antagonism while also revealing potent inhibitory activity against the PAF pathway—suggesting a broader pharmacodynamic role than previously appreciated.

Overall, the data highlight key pathway-specific trends:

- NSAIDs (e.g., lornoxicam, diclofenac) and clopidogrel showed preferential inhibition of the PAF pathway, supporting their dual antiplatelet and anti-inflammatory effects.
- Analgesics and β-blockers (e.g., thiocolchicoside, propranolol) were more selective for the ADP pathway, aligning with a primarily thrombotic-targeted mechanism.
- Antihypertensives (e.g., candesartan, valsartan) demonstrated more balanced activity across both pathways, though efficacy varied among individual agents.

The integration of drug combination testing revealed consistent synergistic enhancement of PAF inhibition, particularly for clopidogrel-based combinations. Toxicological and desensitization assays further identified β-blockers like propranolol as capable of inducing direct, dose-dependent platelet activation, even in the absence of external agonists—highlighting the need for functional platelet assays in safety profiling.

Altogether, these findings underscore the clinical value of dual-pathway screening in drug evaluation, offering mechanistic insight into both thrombotic and inflammatory aspects of cardiovascular pharmacotherapy. The results also call for further investigation into PAF-related drug interactions, a critically underexplored area with major implications for vascular health. Future in vivo and clinical studies are warranted to validate these observations and guide more targeted, multi-pathway therapeutic strategies.

## Data Availability

All data are contained within the article. Any further information concerning raw data or the article can be provided by the authors upon request.

## Supplementary Materials

The following supporting information can be downloaded at: https://www.mdpi.com/article/10.3390/medicina61081413/s1, Table S1: Physicochemical properties of the pharmaceutical compounds evaluated in this study.
