# Supplementary material for: Anti-Inflammatory and Antiplatelet Interactions on PAF and ADP Pathways of NSAIDs, Analgesic and Antihypertensive Drugs for Cardioprotection—In Vitro Assessment in Human Platelets
Source: Medicina (Kaunas). 2025 Aug 4;61(8):1413. doi: 10.3390/medicina61081413 (PMC12388159; doi:10.3390/medicina61081413)
Supplement: Supplementary file 1 [file medicina-61-01413-s001.zip › medicina-3735448-supplementary.pdf]

Supplementary Table S1. Physicochemical properties of the tested compounds.

Bioactives	CAS No	Chemical Structure	Molecular formula	Molecular Weight (g/mol)	pKa	Water solubility	logKow or logP
**Antiplatelet**							
Clopidogrel	113665-84-2	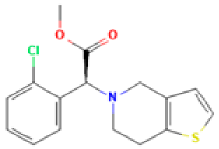	$C_{16}H_{16}ClNO_2S$	321.82	4.77 (basic)	15.1 ug/mL (in buffer at pH 7.4)	3.82
**NSAIDs**							
Diclofenac	15307-86-5	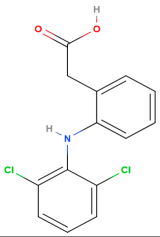	$C_{14}H_{11}Cl_2NO_2$	296.15	4.20 (acidic)	2.37 mg/L (at 25 °C)	4.51
Ketoprofen	22071-15-4	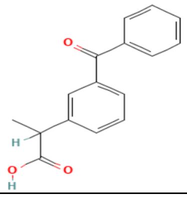	$C_{16}H_{14}O_3$	254.28	4.00 (acidic)	51 mg/L (at 22 °C)	3.12
Lornoxicam	70374-39-9	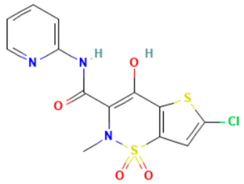	$C_{13}H_{10}ClN_3O_4S_2$	371.81	4.22 (basic), 1.82 (acidic)	15.5 ug/mL (in buffer at pH 7.4)	2.62
Etoricoxib	202409-33-4	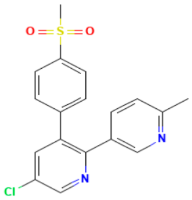	$C_{18}H_{15}ClN_2O_2S$	358.84	4.96 (basic), 19.69 (acidic)	0.0033 g/L	3.70
Nimesulide	51803-78-2	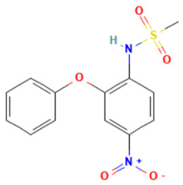	$C_{13}H_{12}N_2O_5S$	308.31	6.70 (acidic), -3.70 (basic)	0.0182 mg/mL	2.60

Niflumic acid	4394-00-7	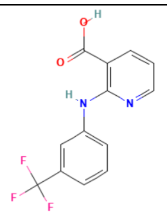	$C_{13}H_9F_3N_2O_2$	282.22	1.88 (acidic), 5.3 (basic)	19 mg/L (at 25 °C)	4.43
Allopurinol	315-30-0	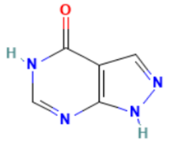	$C_5H_4N_4O$	136.11	8.47 (acidic), 1.25 (basic)	569 mg/L (at 25 °C)	-0.55
Naproxen	22204-53-1	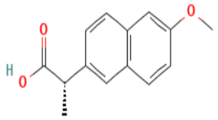	$C_{14}H_{14}O_3$	230.26	4.18 (acidic)	15.9 mg/L (at 25 °C)	3.18
**Analgesics**							
Paracetamol (acetaminophen)	103-90-2	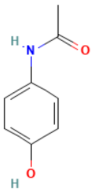	$C_8H_9NO_2$	151.16	9.38 (acidic)	14,000 mg/L (at 25 °C)	0.46
Thiocolchicoside	602-41-5	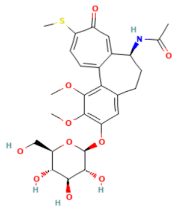	$C_{27}H_{33}NO_{10}S$	563.62	12.20 (acidic), -1.20 (basic)	10 mg/mL	0.34
**β-blockers**							
Propanolol	525-66-6	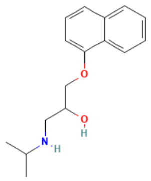	$C_{16}H_{21}NO_2$	259.34	9.42 (basic)	61.7 mg/L (at 25 °C)	3.48
Atenolol	29122-68-7	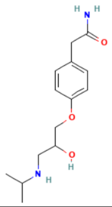	$C_{14}H_{22}N_2O_3$	266.34	9.60 (basic)	13,300 mg/L (at 25 °C)	0.16

Metoprolol	51384-51-1	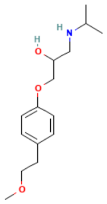	$C_{15}H_{25}NO_3$	267.36	9.56 (basic)	>1,000 mg/mL (at 25 °C)	1.88
Sotalol	3930-20-9	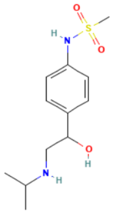	$C_{12}H_{20}N_2O_3S$	272.37	9.76 (basic)	0.78 g/L	1.10
**Antihypertensives**							
Candesartan	139481-59-7	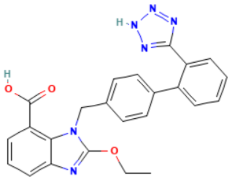	$C_{24}H_{20}N_6O_3$	440.45	2.97 (acidic), 1.71 (basic)	0.0077 g/L	6.10
Valsartan	137862-53-4	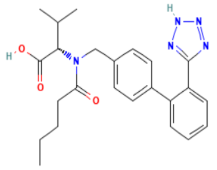	$C_{24}H_{29}N_5O_3$	435.52	4.35 (acidic), -0.64 (basic)	0.023 g/L	4.00
